# EBV Latency Types Adopt Alternative Chromatin Conformations

**DOI:** 10.1371/journal.ppat.1002180

**Published:** 2011-07-28

**Authors:** Italo Tempera, Michael Klichinsky, Paul M. Lieberman

**Affiliations:** The Wistar Institute, Philadelphia, Pennsylvania, United States of America; University of Southern California School of Medicine, United States of America

## Abstract

Epstein-Barr Virus (EBV) can establish latent infections with distinct gene expression patterns referred to as latency types. These different latency types are epigenetically stable and correspond to different promoter utilization. Here we explore the three-dimensional conformations of the EBV genome in different latency types. We employed Chromosome Conformation Capture (3C) assay to investigate chromatin loop formation between the OriP enhancer and the promoters that determine type I (Qp) or type III (Cp) gene expression. We show that OriP is in close physical proximity to Qp in type I latency, and to Cp in type III latency. The cellular chromatin insulator and boundary factor CTCF was implicated in EBV chromatin loop formation. Combining 3C and ChIP assays we found that CTCF is physically associated with OriP-Qp loop formation in type I and OriP-Cp loop formation in type III latency. Mutations in the CTCF binding site located at Qp disrupt loop formation between Qp and OriP, and lead to the activation of Cp transcription. Mutation of the CTCF binding site at Cp, as well as siRNA depletion of CTCF eliminates both OriP-associated loops, indicating that CTCF plays an integral role in loop formation. These data indicate that epigenetically stable EBV latency types adopt distinct chromatin architectures that depend on CTCF and mediate alternative promoter targeting by the OriP enhancer.

## Author Summary

Epstein-Barr Virus (EBV) latent infection is associated with several human malignancies. The viral genes expressed during latent infection can vary depending on host cell or tumor type. The different gene expression programs, referred to as latency types, are determined by alternative viral promoter usage. In this work, we investigate how differential DNA loop formation regulates viral promoter selection in different latency types. We use chromatin conformation capture methods to demonstrate that the transcriptional enhancer at OriP forms a stable loop with one of two different promoters, depending on the latency type. In type I latency, OriP forms a loop with the active Q promoter (Qp). In type III latency, OriP forms a loop with the active C promoter (Cp). Loop formation was mediated, in part, by CTCF binding sites located within the loops. Mutation in the CTCF binding site located at Qp caused a loss of OriP-Qp loop formation, a loss of Qp transcription, and a reactivation of Cp transcription from an alternative loop formed with OriP-Cp. These findings indicate that OriP loop formation is an integral component of promoter selection, and that chromatin conformation may determine EBV latency type.

## Introduction

Epstein-Barr virus (EBV) is a human gammaherpesvirus that is the etiological agent of infectious mononucleosis and is commonly associated with B-cell lymphomas, as well as epithelial malignancies [1], [2]. EBV latent infection is observed in endemic Burkitt's lymphoma (BL), the majority of Hodgkin's lymphomas (HL) and in lymphoproliferative diseases associated with immunosuppression [3]. During latency the EBV genome is maintained as a multicopy, chromatinized episome that can adopt one of four different gene expression patterns. These metastable gene expression patterns are generally referred to as latency types and are classified on the basis of EBNA and LMP protein expression [4]. Type 0 latency is defined as latency with no viral gene expression and is found in non-dividing B-cells [5]. In Type I latency, which is observed in BL and BL-derived cell lines, as well as in memory B-cells in a healthy host, EBNA1 is the only viral gene that is expressed [6], [7], [8], [9], [10]. In undifferentiated nasopharyngeal carcinoma (NPC), EBV-associated gastric carcinoma, HL, and T cell lymphomas EBV expresses LMP1 and LMP2 along with EBNA1 and this pattern is referred to as latency type II [11], [12], [13], [14], [15], [16], [17], [18], [19]. In immunoblastic lymphomas associated with immunosuppression and *in vitro* immortalized lymphoblastoid cell lines (LCLs) EBV is able to express all of the known latency associated genes, EBNA-1, -2, -3A, -3B, -3C, -LP, LMP1 and LMP2 [20]. These different patterns of gene expression are epigenetically stable, in that they are maintained over multiple cell divisions, and generally reflect developmental fate-associated properties of the host-cell.

Latency type gene expression patterns typically correspond to distinct primary transcripts that are initiated from different viral promoters [21], [22], [23], [24]. In type I latency EBNA1 is transcribed starting from Qp in the BamHI/Q region of the EBV genome, while in type III latency the polycistronic mRNA for EBNA proteins is initiated from Cp located in the BamHI C region of the EBV genome [22], [24], [25], [26]. Promoter selection is a critical step for establishing which gene expression program EBV adopts. Both viral and cellular transcription factors are known to regulate these alternative promoters [27], yet many questions still remain.

For example, EBNA1 binding sites at OriP serve as an essential enhancer of Cp in the establishment of type III latency, but it is not known how this enhancer physically targets Cp rather than Qp [28], [29], [30]. It is also known that EBNA1 binding to the Qp transcription initiation site inhibits Qp transcription in type III latency [31], but it is not known how this repression is reversed during the transition to type I latency. Furthermore, epigenetic modifications are known to play an important role in regulating promoter selection [32], [33]. In type I latency Cp is enriched for deacetylated histones and hypermethylated DNA, while Qp is associated with acetylated histones and elevated H3meK4 [34], [35], [36], [37]. In contrast, in type III latency H3meK4 levels are high at Cp along with histone acetylation and DNA hypomethylation while Qp lacks histone modifications associated with active transcription [35], [37]. How these DNA binding factors and chromatin modifications orchestrate the mutually exclusive alternative promoter selection of Cp or Qp remains an important unanswered question for understanding EBV latency control mechanisms.

Several recent findings have revealed that higher-ordered chromosome structures, including DNA loops, chromatin domains, and insulators, play an important role in gene expression programming [38], [39]. The use of new techniques such as Chromosome Conformation Capture (3C) and variations of this assay revealed a complex and diffuse network of long-distance interactions between DNA regions both intra- and inter-chromosomal [40], [41], [42], [43]. Many of these chromatin loops are dynamic and may reflect alternative genome packaging into the nucleus, while other interactions may reflect programmed long-distance interactions that play a crucial role in gene regulation. Identification of cellular factors that regulate loop-formation and long distance interactions support the importance of three-dimensional structures in regulating gene expression [44], [45].

The CCCTC-binding factor (CTCF) has been reported in several studies to be involved in promoting and regulating chromatin loop formation [46], [47], [48], [49], [50]. CTCF is a nuclear DNA binding protein that contains eleven zinc fingers and is well conserved among higher eukaryotes [51], [52], [53]. CTCF is involved in different functions including chromatin boundary formation, DNA loop formation, transcriptional activation and repression, and promoter-enhancer blocking activity [46], [47]. In EBV, CTCF binds at several key regulatory regions [35]. In particular, CTCF can bind between OriP and Cp to negatively regulate transcription initiating at Cp in type I latency [54]. In addition, CTCF can bind upstream of Qp, and this binding is crucial for maintaining transcription activation and protecting Qp from epigenetic silencing [37]. However, the role of CTCF in EBV chromatin conformation has not been addressed. Here, we use 3C and variations of 3C to probe the conformations of the EBV latency control regions in different latency types.

## Results

### 3C analyses of EBV type I and type III genomes reveal distinct conformations of the OriP enhancer region

**Figure 1:**
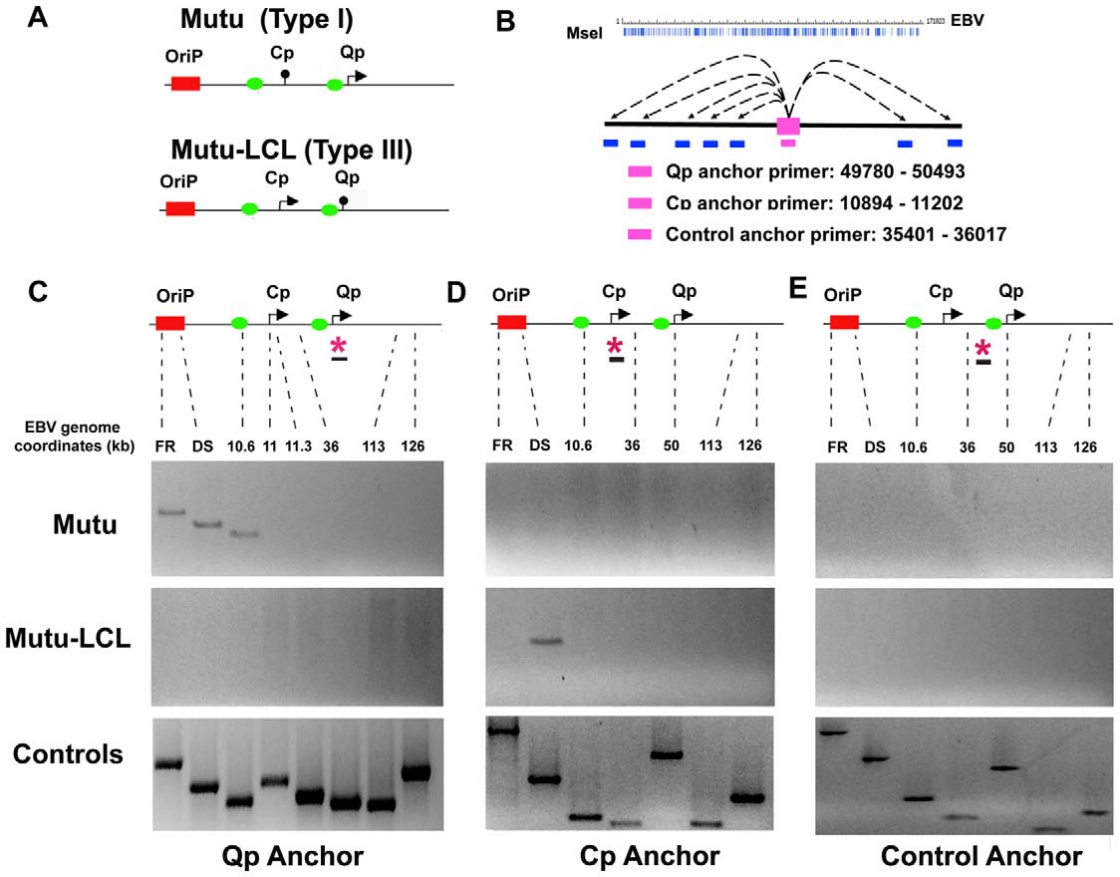
OriP region forms alternative chromatin loops in type I and type III latency types. A) Schematic of type I and type III promoter usage and relative position of OriP, Cp, and Qp are indicated. Green boxes indicate known CTCF binding sites. B) Schematic of MseI restriction site map and the anchor primers used for Qp, Cp, and control. EBV genome coordinates for each anchor primer are indicated. C) 3C PCR analysis using the Qp anchor primers and several acceptor regions of EBV genome for FR, DS, 10.6, 11 11.3, 36, 113, and 126 kbp. Representative gel images for Mutu I (top), Mutu-LCL (middle) or control bacmid ligation mixtures (lower) are indicated. D) Representative gel images of 3C PCR analysis using the Cp anchor primer and several acceptor regions of the EBV genome as indicated. E) Representative gel images of 3C PCR analysis using the anchor primer within the fragment chosen as control and several regions of the EBV genome, as indicated.

To determine if the differences in EBV latency type gene expression correlate with differences in three-dimensional organization of the EBV episome, we employed the 3C assay (Fig. 1). EBV positive B-cell lines with stable type I (Mutu I) or type III (Mutu-LCL) latency types were cross-linked with formaldehyde for 30 min and then processed for 3C after fragmentation with MseI restriction enzyme, which generates 342 fragments ranging from 4 bp to ∼4 kbp (Fig. 1A and B). Primers located in MseI fragments containing Cp (10894–11202) or Qp (49780–50493) were used as anchors for the PCR analysis of 3C interactions with several other regions of the EBV genome, including FR, DS, and the CTCF binding site at position 10.6 kb situated between DS and Cp [54]. A third anchor primer at position 35401–36017 was used as a control for 3C product specificity (Fig. 1B). As a positive control for primer efficiency we used a random ligation mixture of MseI digested EBV bacmid DNA in order to contain every possible combination of ligation products.

The Qp promoter is active in type I latency, and silenced in type III latency (Fig. 1A). When Qp anchor primers were used for 3C, we observed a strong ligation product between Qp and the OriP region in Mutu (Type I) (Fig. 1C, top panel). Interestingly, Qp interactions were identified for both DS (∼9000) and FR (∼7000) elements of OriP. Qp also interacted with the CTCF-binding site upstream the Cp promoter (∼10,600). Qp did not form detectable 3C ligation products with several other regions of the EBV genome, including regions at 11, 11.3, 36, 113 and 126 kb on the EBV genome. In contrast to type I latency, Qp did not form any 3C products in type III latency (Mutu-LCL), where Qp is transcriptionally silent (Fig. 1C, middle panel). These findings indicate that the 3C ligation products between Qp and OriP region correlate with transcription activity of Qp. All ligation products could be detected in control reactions with bacmid DNA (Fig. 1C, lower panel). These findings suggest that Qp forms specific DNA looping interactions with OriP and the CTCF binding site upstream of Cp in type I cells, but not in type III cells.

We next examined the three dimensional architecture of Cp in both type I and type III (Fig. 1C). In type I latency (Mutu I), where Cp is transcriptionally silent, the Cp anchor failed to form any detectable 3C products with acceptor primers (Fig. 1D, top panel). In type III latency (Mutu-LCL), where Cp is transcriptionally active, we found a predominant 3C interaction between Cp and the DS element of OriP (Fig. 1D, middle panel). All primer sets were capable of amplifying ligation products from control bacmid DNA (Fig. 1D, lower panel).

To verify that 3C interactions between Qp or Cp with OriP were specific and not due to a generic conformation of OriP in type I or type III, we used a primer at 35401 (a region equidistant from both Cp and Qp) as an anchor for the PCR analysis (Fig. 1E). The 35401 anchor primer was unable to form 3C interactions with OriP or any other region of the EBV genome assayed in type I (Fig. 1E, top panel) or type III (Fig. 1E, middle panel) cells. All primer sets were capable of generating PCR products from control ligation reactions with EBV bacmid DNA (Fig. 1E, lower panel). Furthermore, all major 3C products were gel purified, subjected to direct DNA sequencing, and validated for correct ligation products (data not shown). These finding indicate that the 3C products formed between Qp and OriP in type I, and Cp and OriP in type III cells, are sequence and cell-type specific interactions.

**Figure 2:**
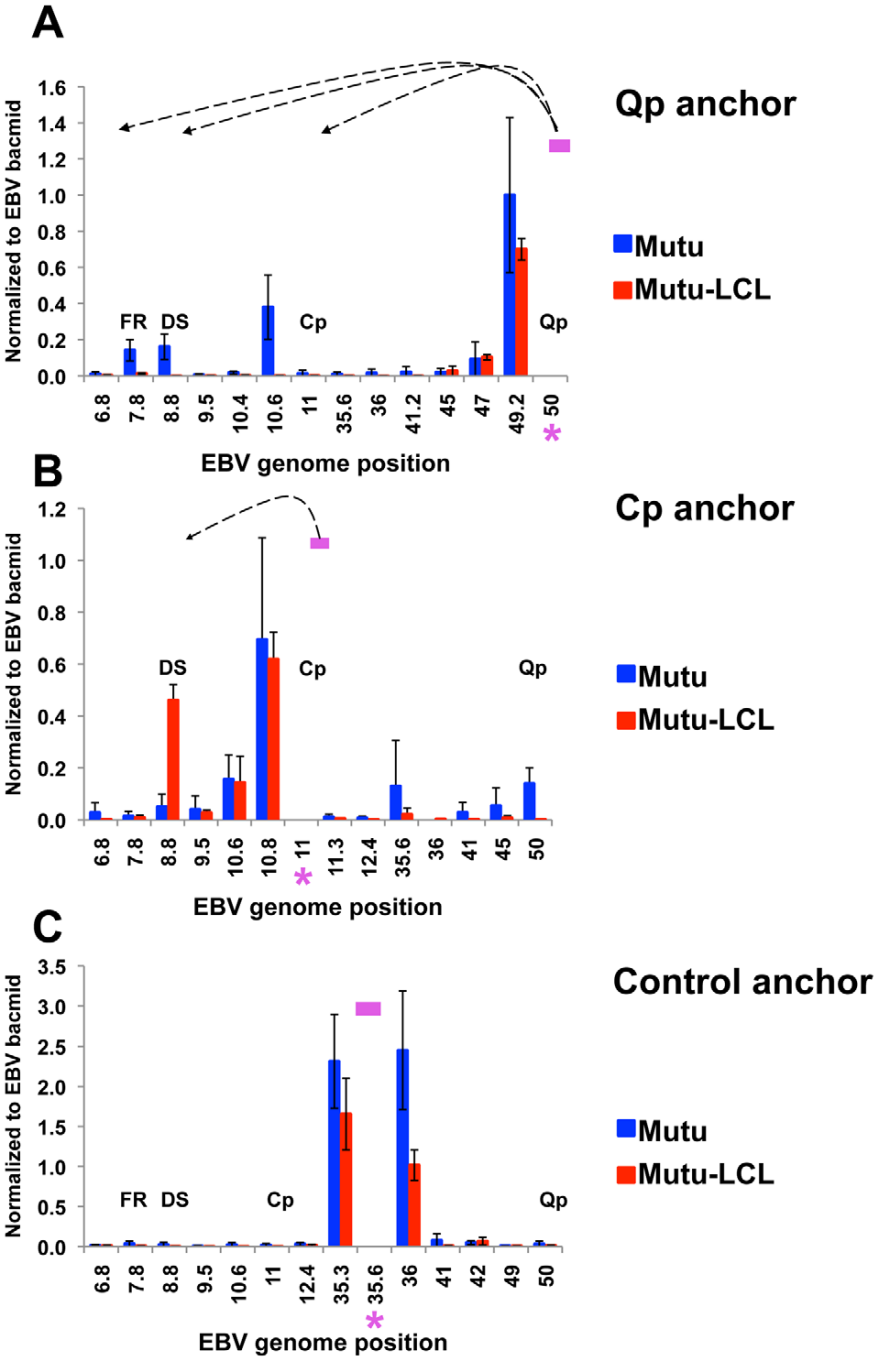
Quantitative 3C analysis confirms alternative loop formation between type I and type III latency types. 3C assay coupled with quantitative PCR was used to analyze chromatin architecture of the EBV genome in type I (Mutu) and type III (Mutu-LCL) latencies. EBV genome coordinates of all the Mse I fragments tested are indicated on the X-axis. A pink asterisk indicates fragment bearing the anchor primer. Loop formation between the anchor primer (pink box) and an Mse I fragment is indicated by dashed arrows. Data were analyzed using the ΔCt method, and Ct values generated by using 100 ng of EBV bacmid DNA Mse I digested and randomly ligated were used as the control. Data were obtained by three independent experiments and expressed as mean ± SE. Data generated by the anchor fragment self-ligation product were removed from analysis. (A) Quantitative 3C analysis using the anchor primer within the fragment containing Qp and several regions of the EBV genome. (B) Quantitative 3C analysis using the anchor primer within the fragment containing Cp and several regions of the EBV genome. (C) Quantitative 3C analysis using the anchor primer within the fragment chosen as control and several regions of the EBV genome.

To validate the conventional PCR data and to better quantify the 3C interaction products, we analyzed the chromatin conformation by real time PCR using a different set of primer pairs (Fig. 2). When Qp anchor primers were assayed, interactions with OriP (FR and DS) and CTCF-Cp binding site (10.6) were observed in type I (Mutu), but not in type III (Mutu-LCL) (Fig. 2A). When Cp anchor primers were used, an interaction with DS was detected in type III (Mutu-LCL), but not in type I (Mutu) (Fig. 2B). The control anchor at 35.6kb failed to form any specific 3C products in type I or type III cells (Fig. 2C). In all cases, a strong interaction can be detected at regions immediately adjacent to the anchor primers, which is expected in quantitative 3C reactions, but does not indicate a specific chromatin conformation [55]. The specificity of the primer set was tested by agarose gel electrophoresis and dissociation curve analysis (Figures S1-4).

### OriP binding factors EBNA1, ORC2 and TRF2 interact with active promoters for each latency type

**Figure 3:**
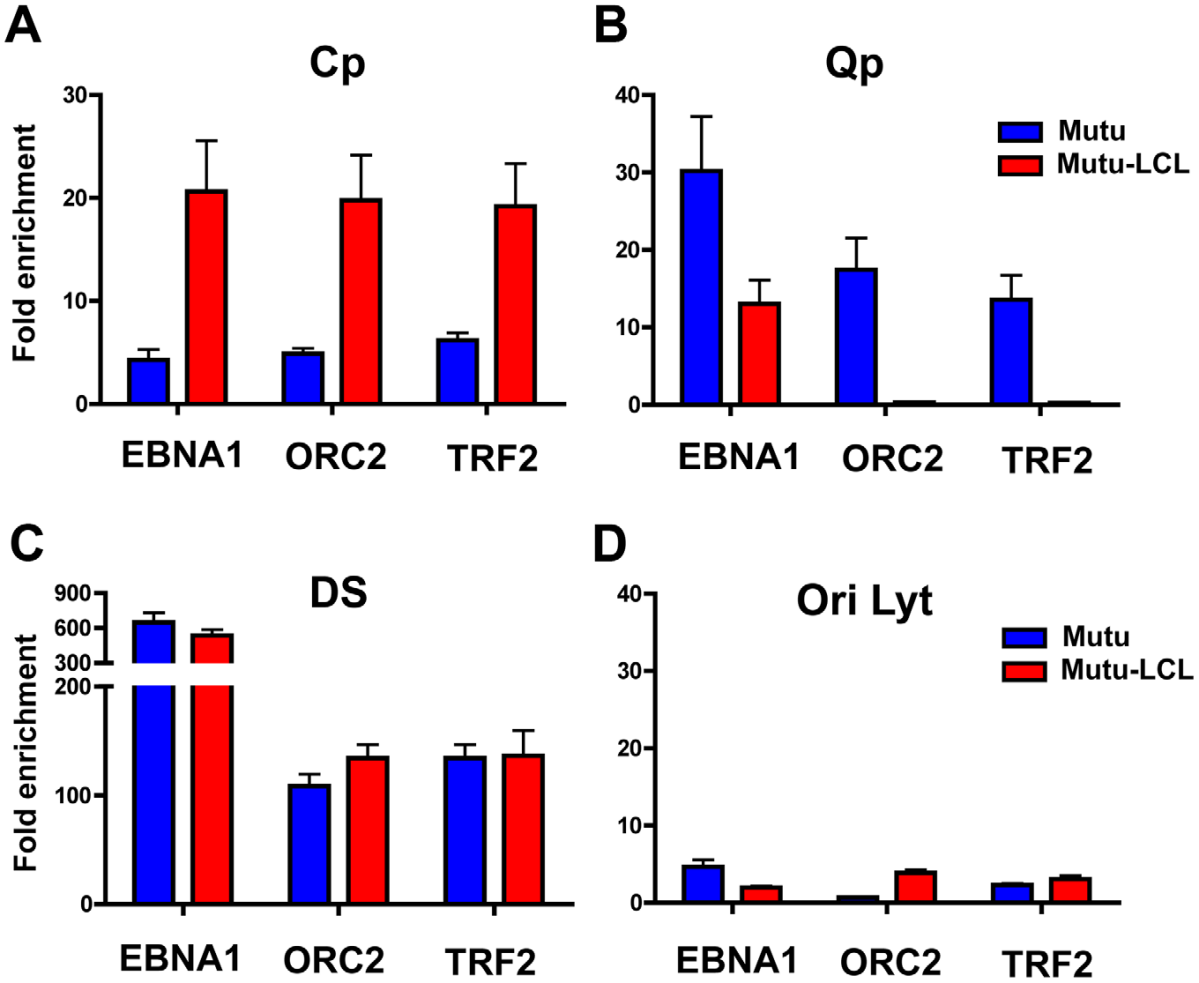
Interaction between OriP binding factors and promoters correlates with promoter activation. ChIP-qPCR analysis of EBNA1, ORC2, and TRF2 at target regions in type I (Mutu, blue) and type III (Mutu-LCL, red) type latency. Target regions were Cp (panel A), Qp (panel B), DS (panel C), and OriLyt (panel D), as indicated. ChIP-qPCR was performed in conditions that preserved chromatin loop formation. Each bar represents the mean ± SE of three independent experiments. Data are expressed as fold enrichment of specific factor binding relative to that of an IgG control.

To validate the presence of a chromatin loop between OriP and either Cp or Qp promoters in their respective latency types, we used a modified Chromatin Immunoprecipitation (ChIP) assay (Fig. 3). We reasoned that OriP binding factors should associate with either Cp or Qp if they were in close physical proximity due to a DNA loop between these different DNA loci. To measure these potentially indirect protein-DNA interactions, we increased the crosslink time as long as for 3C (30 min) in order to preserve any chromatin loop formation. Previous studies have demonstrated that cellular proteins TRF2 and ORC2, and the viral protein EBNA1 bind at the DS region of OriP in both type I and type III cells [56], [57]. We therefore assayed TRF2, ORC2, and EBNA1 for interactions with either Qp or Cp using this extended cross-linking ChIP assay. The DNA was analyzed by real time PCR using primers specific for different regions of the EBV genome. We found a significant enrichment of ORC2, TRF2 and EBNA1 at Cp in type III but not in type I (Fig. 3A), suggesting that the OriP region is in close proximity to the active (type III) Cp, in agreement with the 3C data. In contrast, ORC2 and TRF2 were enriched at Qp only in type I cells, where Qp is active (Fig. 3B). EBNA1 was able to bind at Qp both in type I and type III, consistent with the known sequence-specific binding of EBNA1 at Qp. It is worth noting that Qp is ∼40 kb from the OriP region and that these ChIP results are consistent with the chromatin loop observed in the 3C assay. As a positive control we tested the enrichment of all three proteins at the DS region of OriP (Fig. 3C). We found that EBNA1, ORC2, and TRF2 bound with high occupancy (>100 fold enrichment) at DS in type I and type III latencies. To rule out any non-specific binding caused by the increase in cross-linking time we evaluated the binding profile for all three proteins at OriLyt (Fig. 3D). We did not detect any significant enrichment for EBNA1, ORC2, or TRF2 with OriLyt in either latency type, indicating that the extended cross-linking time did not cause non-specific ChIP interactions.

### CTCF is involved in chromatin loop formation in EBV latency types

**Figure 4:**
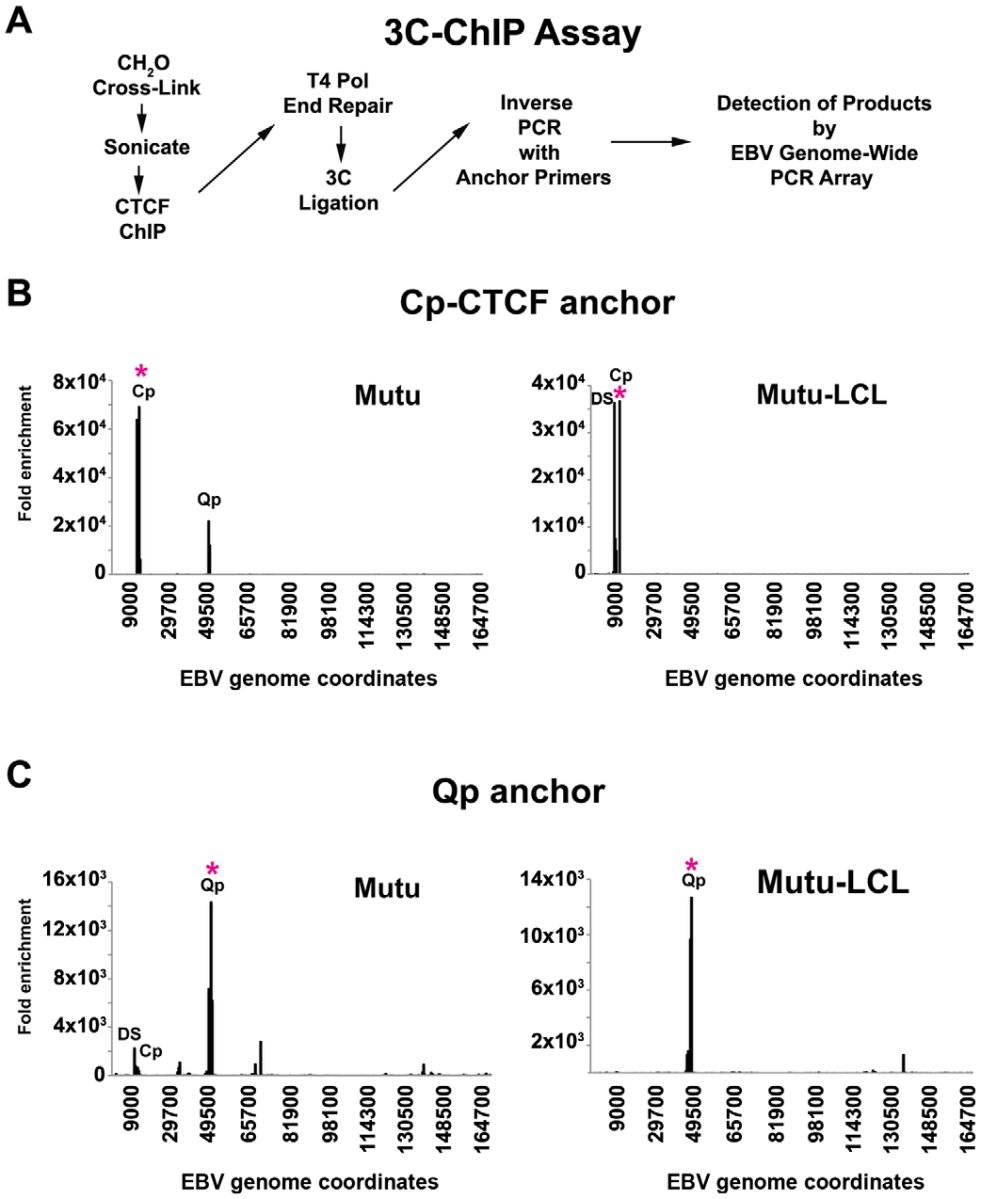
Chromosome Conformation Capture Chromatin Immunoprecipitation (3C-ChIP) analysis shows that CTCF is involved in chromatin looping in EBV. A) Schematic description of the 3C-ChIP assay used for analysis of CTCF mediated interactions with the EBV genome. B and C) 3C-ChIP assay using either Cp-CTCF anchor primers (panels B) or Qp anchor primers (panels C) with Mutu (left panel) or Mutu-LCL (right panel). Anchor primer regions are indicated by an asterisk. EBV genome coordinates of EBV 384-well genome array are indicated on the X-axis. Data are representative of two independent experiments and are expressed as fold enrichment of CTCF binding over that of an IgG control. Only data with a SD less then 10% were considered.

CTCF can bind upstream of both Cp and Qp, and influence their transcription activity [37], [54]. Since CTCF has been implicated in DNA looping and chromatin organization, we set out to determine if CTCF was involved in chromatin loop formation in EBV. To address this, we developed a new method, which we refer to as 3C-ChIP, that combines 3C and ChIP to address the role of a specific factor in mediating 3C linkages (Fig. 4A). Specifically, cells were subject to formaldehyde cross-linking for 30 min, followed by sonication and ChIP assay with CTCF-specific antibody. The ChIP DNA was then end-repaired by T4 DNA polymerase and ligated to its nearest cross-linked neighbor. Anchor regions for CTCF sites at Qp (49500) or Cp-CTCF (10350) were then used to design inverse PCR primers to amplify cross-linked DNA by circular inverse PCR. The inverse PCR-products were then analyzed by real time PCR using a 384-well array that covers the entire genome of EBV. When Cp-CTCF was used as the anchor in type I cells, we observed a peak at Cp and another one at Qp (Fig. 4B left panel). The peak at Cp-CTCF is mostly the self-ligated product generated during PCR amplification. The peak at Qp suggests that a chromatin loop exists between this region and the CTCF-binding site upstream of Cp. In type III (Mutu-LCL) cell lines, we detected the self-ligation product with Cp-CTCF, but also a strong peak at DS in the OriP region, consistent with the loop observed from 3C data in type III latency (Fig. 4B right panel). When Qp was used as the anchor, a large self-ligation peak appeared, as expected (Fig. 4C). In addition to the self-ligation, interactions between Qp and DS, and to a lesser extent with Cp, were revealed in type I cells (Mutu) (Fig. 4C, left panel). We also observed several minor peaks in the regions around ∼35500, ∼68000 and ∼138000 bp of the EBV genome. Interestingly, these regions all correspond to CTCF binding sites, which were mapped in a previous study [37]. In contrast, the Qp anchor region produced only self-ligation in type III cells (Mutu-LCL) (Fig. 4C right panel). None of the 3C-ChIP peaks were observed when the cross-linking step was eliminated from the protocol, indicating that these products are strictly dependent on CTCF interactions (Fig. S5). These data support the conclusion that DNA interactions between Qp and Cp are distinct in different latency types, and that these interactions may be mediated by CTCF.

### The CTCF binding site at Qp is required for chromatin loop formation with OriP

**Figure 5:**
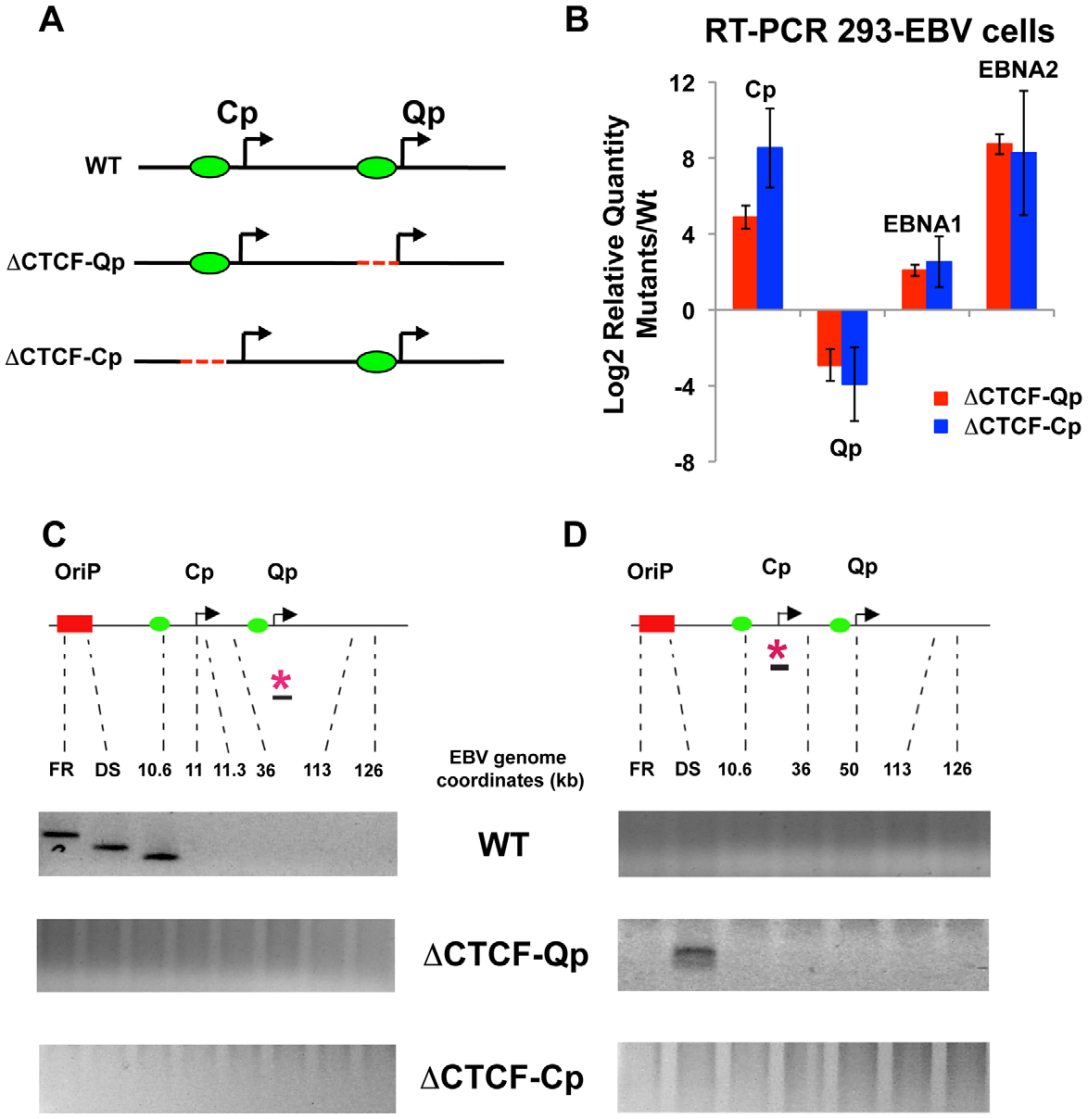
Mutation in the CTCF binding site at Qp and at Cp alters chromatin conformation and promoter activation. (A) Schematic representation of the EBV genome around the Cp and Qp promoters in the Wt and mutant bacmids. A red dashed line represents mutations that abolish CTCF binding. Green circles indicate known CTCF binding sites. (B) Analysis of mRNA expression for EBNA1 and EBNA2 gene and promoter utilization by RT-qPCR. Data are normalized to GFP protein and expressed as log_2_2^ΔΔC t^. Each bar represents the mean ± SE of three independent experiments. (C) Representative gel images of 3C PCR analysis using the anchor primer within the fragment containing Qp promoter and several regions of EBV genome for Wt (top panel) or ΔCTCF-Qp (middle panel) or ΔCTCF-Cp (lower panel). (D) Representative gel imagine of 3C PCR analysis using the anchor primer within the fragment containing Cp promoter and several regions of the EBV genome for Wt (top panel) or ΔCTCF-Qp (middle panel) or ΔCTCF-Cp (lower panel).

To better study the role of CTCF in loop formation we employed EBV bacmids carrying mutations for the CTCF binding site at Qp and Cp. (Fig. 5A). We have previously shown that the mutations disrupting CTCF binding at either Qp or Cp alter viral transcription control and promoter selection in 293 cells [37], [54]. We therefore introduced the Wt, the ΔCTCF-Qp and the ΔCTCF-Cp bacmid in 293 cells and generated a stable cell line by selecting for hygromycin resistance and GFP expression. Consistent with a previous study, both ΔCTCF-Qp and ΔCTCF-Cp cells adopted a type III latency type expression profile with high levels of EBNA2 expression, Cp promoter activation, and Qp transcription repression, while Wt Bacmids adopted a type I latency expression pattern, which is typical of EBV in 293 cells [58] (Fig. 5B and S6). To examine the contribution of CTCF at Qp and Cp on EBV chromatin architecture, we compared Wt and ΔCTCF genomes using 3C assays (Fig. 5C and D). In Wt cells, we found that Qp is able to establish a strong interaction with OriP and CTCF-Cp (Fig. 5C, top panel), similar to the loop observed in Mutu I cell lines (Fig. 1). Interestingly, in ΔCTCF-Qp and ΔCTCF-Cp cells, loop formation was not detected between Qp and either OriP or CTCF-Cp binding site (Fig. 5C, middle and lower panels). When Cp was used as anchor primer, loop formation was detected between Cp and DS in ΔCTCF-Qp cells (Fig. 5D, middle panel), similar to the loop that was observed in type III Mutu LCL (Fig. 1). This interaction was not detected in Wt bacmid in 293 cells (Fig. 5D, top panel), consistent with Wt cells having a type I phenotype. Surprisingly, Cp was not able to form any loop in ΔCTCF-Cp cells (Fig. 5D, lower panel), suggesting that CTCF at Cp is necessary for the long-distance interaction with OriP. However, loss of CTCF binding had a net positive effect on Cp transcription, perhaps reflecting the ability of CTCF to insulate OriP enhancer activation at Cp. Control anchor primers (35401–36017) showed no significant loop formation in these assays, indicating that these 3C interactions are specific (Fig. S7).

**Figure 6 ppat-1002180-g006:**
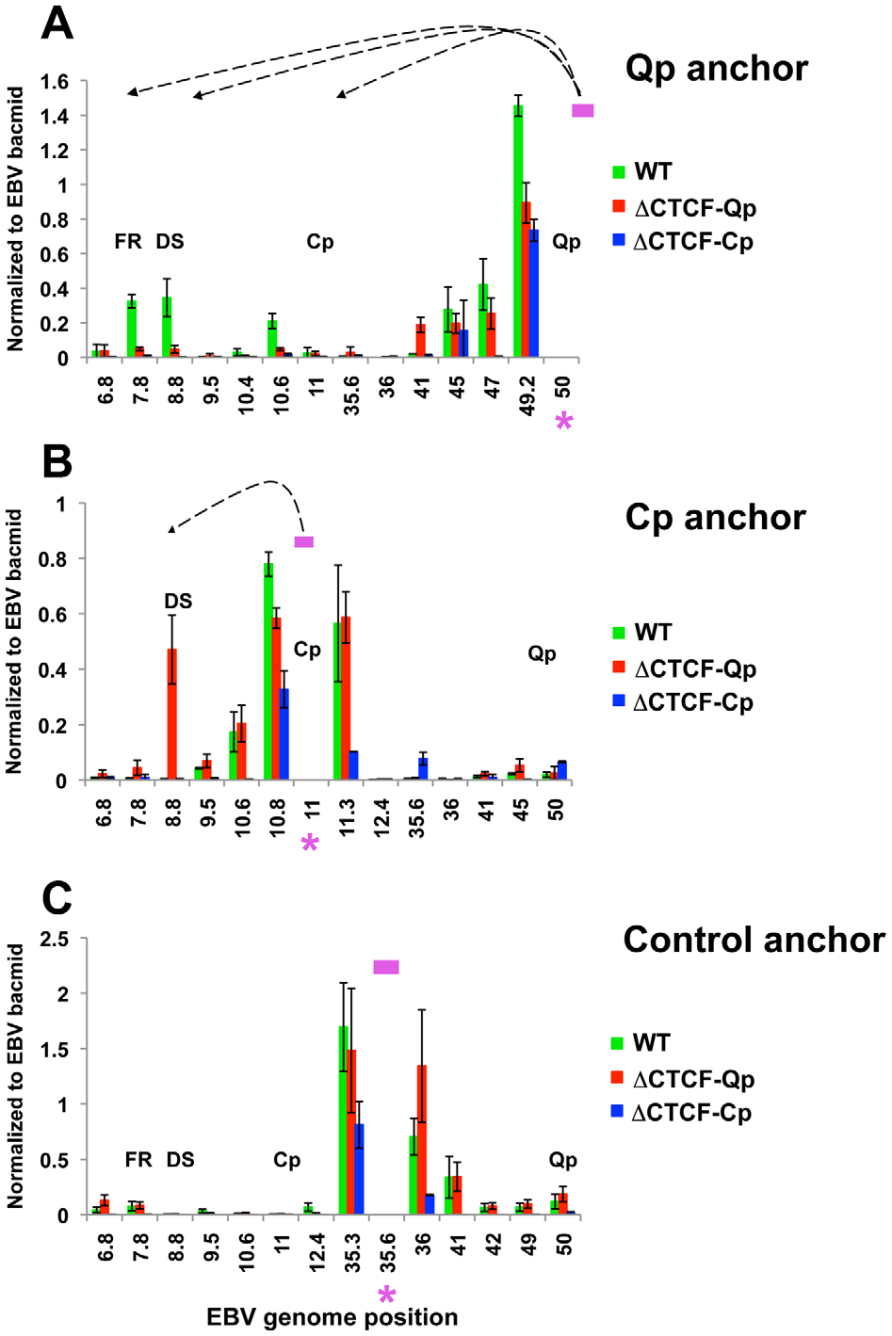
Quantitative 3C analysis confirms changes in chromatin architecture in ΔCTCF-Qp and ΔCTCF-Qp compared to Wt EBV bacmid. 3C assay coupled with quantitative PCR was used to analyze EBV chromatin architecture in 293-EBV Wt bacmid, 293-EBV ΔCTCF-Qp and 293-EBV ΔCTCF-Cp stable cell lines. EBV genome coordinates of all the MseI fragments tested are indicated on the X-axis. A pink asterisk indicates the fragment bearing the anchor primer. Loop formation between the anchor primer (pink box) and a MseI fragment is indicated by dashed arrows. PCR amplification was analyzed using the ΔCt method, and Ct values generated by using 50 ng of EBV bacmid DNA Mse I digested and randomly ligated were used as the control. Data were obtained by three independent experiments and expressed as mean ± SE. Data generated by the anchor fragment self-ligation product were removed from analysis. (A) Quantitative 3C analysis using the anchor primer within the fragment containing Qp and several regions of the EBV genome. (B) Quantitative 3C analysis using the anchor primer within the fragment containing Cp and several regions of EBV genome. (C) Quantitative 3C analysis using the anchor primer within the fragment chosen as control and several regions of the EBV genome.

These 3C assays were repeated using different primer sets for more quantitative real-time PCR analysis of the 3C products (Fig. 6). Analysis of the Qp anchor interaction pattern confirmed the loop formation between OriP and the CTCF-Cp binding site in Wt EBV bacmid containing cell lines, but not in ΔCTCF-Qp and ΔCTCF-Cp bacmid genomes (Fig. 6A). Interestingly, in ΔCTCF-Qp cell lines a weak interaction was observed with the region at 41000 (Fig. 6A). Analysis of the Cp anchor region showed interactions between the Cp region and OriP in ΔCTCF-Qp but not in the Wt or ΔCTCF-Cp (Fig. 6B). The control anchor primer did not form any significant peaks at OriP or CTCF-Cp or Qp (Fig. 6C). These data suggest that CTCF binding at Qp and at Cp is important for directing chromatin looping between Qp and OriP, as well as restricting loop formation between Cp and OriP in 293 cell lines. Moreover, loop formation is correlated with promoter transcription activation at Qp.

### OriP binding factors EBNA1, ORC2, and TRF2, interact with Wt-Qp but not ΔCTCF-Qp

**Figure 7:**
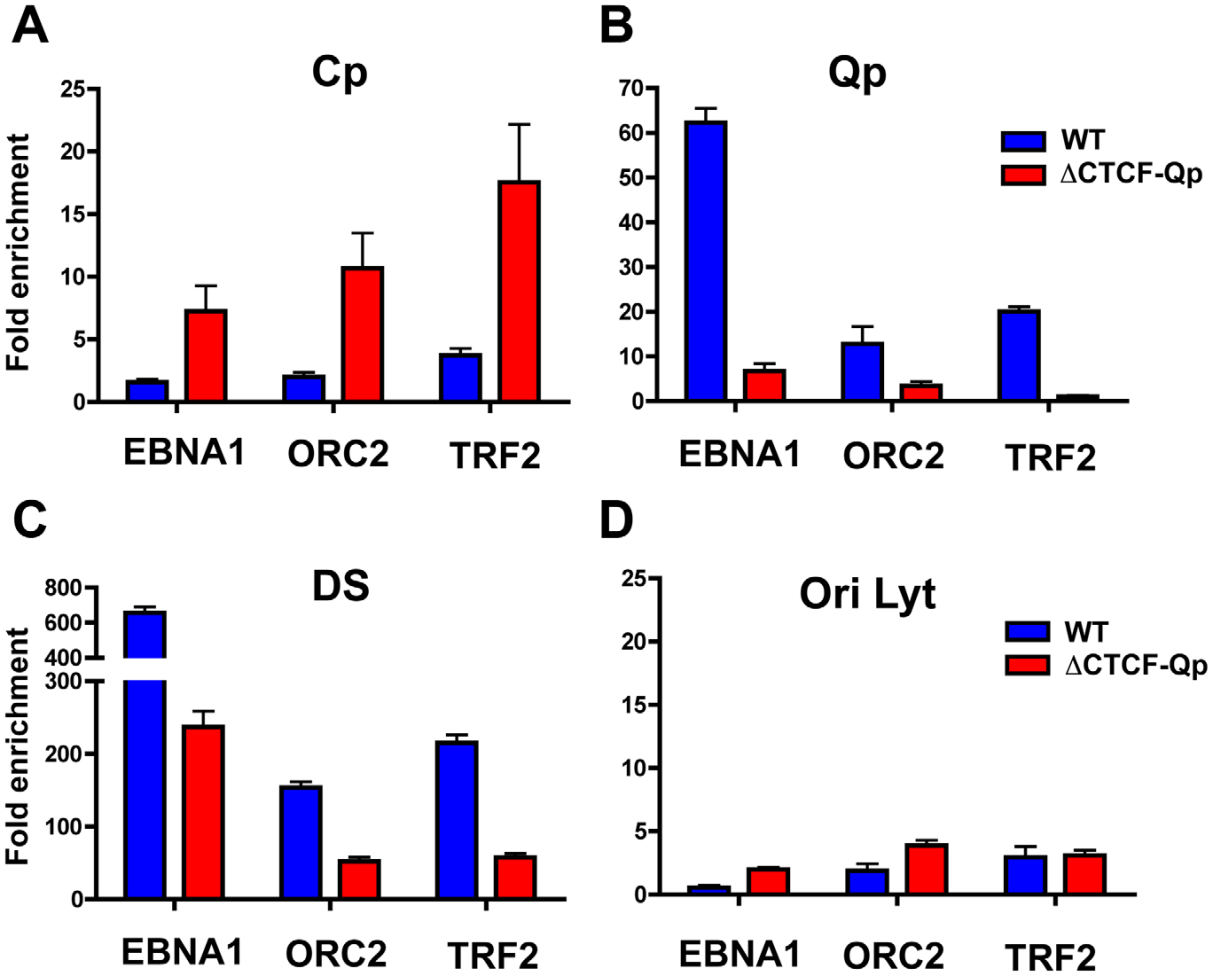
Absence of chromatin loop formation correlates with a loss of interaction between OriP binding factors ORC2 and TRF2 at Qp. ChIP-qPCR analysis of EBNA1, ORC2 and TRF2 at target regions in Wt and ΔCTCF EBV-positive 293 stable cell lines. ChIP-qPCR was performed in conditions that preserved chromatin loop formation. Each bar represents the mean ± SE of three independent experiments. Data are expressed as fold enrichment of specific factor binding relative to that of an IgG control. Target regions used for PCR amplification were Cp (panel A), Qp (panel B), DS (panel C), and OriLyt (panel D).

We next investigated if OriP binding factors, EBNA1, ORC2 and TRF2, can interact with either Cp or Qp through loop formation in the EBV bacmids by using an extended cross-linking method for the ChIP assay (Fig. 7). We found that EBNA1, ORC2, and TRF2 were enriched at Cp in ΔCTCF genomes, but not in Wt cells (Fig. 7A), consistent with Cp activation in ΔCTCF cells. In contrast, EBNA1, ORC2, and TRF2 were enriched at Qp in Wt relative to ΔCTCF cells (Fig. 7B), consistent with Qp activation in Wt cells. EBNA1 binding at Qp was also reduced in ΔCTCF genomes, suggesting that CTCF can affect EBNA1 binding at Qp. We also evaluated the binding of EBNA1, ORC2, and TRF2 at DS (Fig. 7C). While EBNA1, ORC2, and TRF2 bound DS in Wt cells, binding was reduced in ΔCTCF-Qp cells, perhaps reflecting cooperative interactions between Qp and DS mediated by CTCF and chromatin looping. No significant binding of EBNA1, ORC2, or TRF2 was detected at the negative control OriLyt region (Fig. 7D). We also confirmed that CTCF binding at Qp was enriched in Wt and eliminated in ΔCTCF-Qp genomes, as expected (data not shown). These findings support the model that CTCF binding at Qp mediates interactions with OriP in type I latency.

### Depletion of CTCF affects the three-dimensional organization of the EBV genome

**Figure 8:**
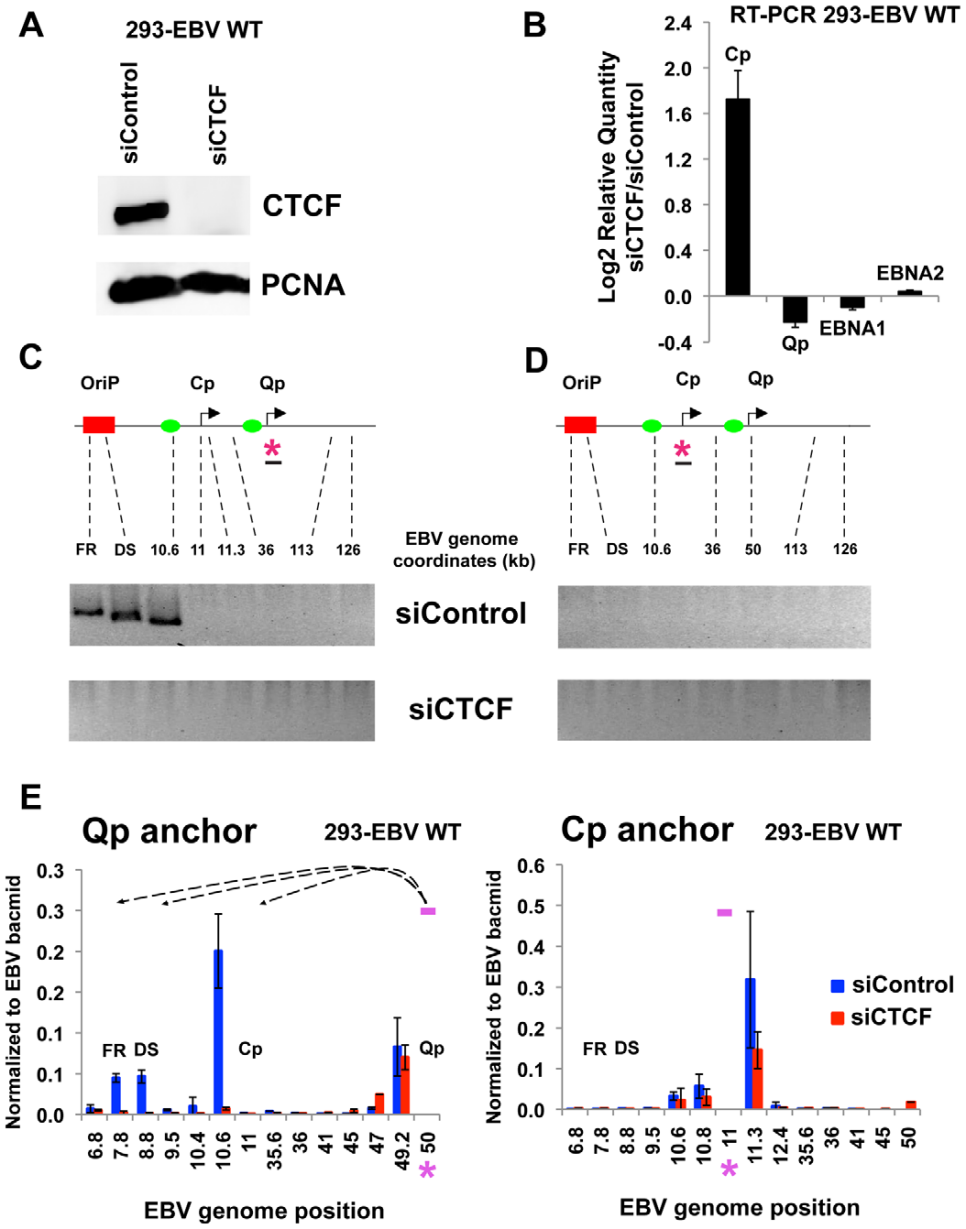
CTCF siRNA depletion disrupts chromatin architecture of EBV. (A) 293-EBV Wt cell lines were transfected with siRNA against CTCF (siCTCF) or a non-targeting control (siControl). After 72 hrs cells were harvested and then analyzed by western blot with antibody to CTCF (top panel) or loading control PCNA (lower panel) to check depletion efficiency. (B) RT-qPCR analysis of promoter utilization and EBNA1 and EBNA2 gene expression after CTCF depletion. Data are normalized to GFP mRNA and expressed as −ΔΔCt values. Each bar represents the mean ± SE of three independent experiments. (C and D) 3C assay was used to analyze chromatin conformation after depletion of CTCF. Representative gel images of 3C PCR analysis using the anchor primer within the fragment containing Qp promoter (C) or Cp promoter (D) and several regions of the EBV genome for 293-EBV Wt cell line transfected with siControl or siCTCF siRNA. Amplified regions are indicated above each gel. (E) 3C assay coupled with quantitative PCR was used to analyze EBV chromatin architecture in 293-EBV Wt bacmid transfected with siControl or siCTCF siRNA, using the anchor primer within the fragment containing Qp promoter (left panel) or Cp promoter (right panel) and several regions of EBV genome. EBV genome coordinates of all the Mse I fragments tested are indicated on the X-axis. A pink asterisk indicates fragment bearing the anchor primer. Loop formation between anchor primer (pink box) and a Mse I fragment is indicated by dashed arrows. PCR amplification was normalized by using 50 ng of EBV bacmid DNA Mse I digested and randomly ligated. Data were obtained by three independent experiments and expressed as mean ± SE. Data generated by the anchor fragment self-ligation product were removed from analysis.

To determinate if CTCF levels in cells can influence chromatin loop formation 293-EBV Wt cell lines were transfected with siRNA against CTCF (siCTCF) or non-targeting control (siControl). We used 293-EBV positive cell lines because of their high transfection efficiency compared to human B cells (Fig. 8). siCTCF transfection completely depleted CTCF levels in 293-EBV Wt cells compared to siControl (Fig. 8A). By real time RT-PCR we investigated the effect of siCTCF transfection on promoter utilization and viral genome expression (Fig. 8B). We observed that CTCF depletion caused a moderate increase in Cp activation and a slight decrease in Qp transcription. However, neither EBNA1 nor EBNA2 mRNA levels were significantly affected by siCTCF. 3C analysis was then carried out to determine the effect of siCTCF on chromatin architecture. In 293-EBV Wt cells transfected with siControl we were able to observe chromatin loop formation between Qp and OriP (Fig. 8C, top panel). Interestingly, the chromatin loop between Qp and OriP was completely disrupted in siCTCF transfected cells (Fig. 8, lower panel). Consistent with previous observations, no chromatin loop was detected when Cp was used as the anchor primer in these EBV-293 cell lines (Fig. 8D). 3C assay coupled with real time PCR analysis was then employed to better quantify our analysis (Fig. 8E). Real time PCR confirmed that Qp-OriP interaction was significantly reduced in siCTCF but not in siControl transfected cells (Fig. 8E), left panel. No significant peaks were observed when the Cp region was used as anchor primer (Fig. 8E, right panel). The control anchor primer did not form any significant chromatin loops with OriP or Cp or Qp regions (Fig. S8). These data indicate that CTCF is critical for the establishment and maintenance of the OriP-Qp chromatin loop in EBV-transfected 293 cell lines.

## Discussion

**Figure 9:**
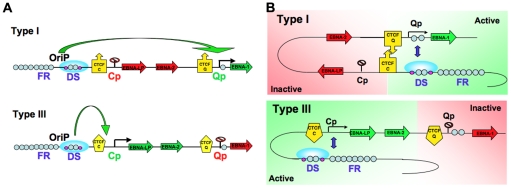
Model of chromatin conformational control of EBV latency types. A) Linear model of EBV latency types depicting OriP enhancer interactions with Cp (type III) or Qp (type I). CTCF binding sites are shown as a yellow box with an arrow. B) Conformational model showing OriP recruiting Qp into an active loop and domain in type I cells (top panel), and the alternative conformation where OriP recruits Cp into a loop and active domain in type III cells (lower panel).

Chromatin structure is known to play an important role in gene expression by regulating the accessibility of the DNA to transcription factors and RNA polymerase II. Emerging evidence shows that not only the composition of the chromatin fiber, but also the three-dimensional structure of chromatin can influence gene transcription. We and others have shown that epigenetic features, including chromatin structure, play an important role in regulating EBV gene expression during latency [21], [34], [37], [54], [59]. We have also reported that the chromatin-organizing factor CTCF binds at Cp and Qp, and is important for maintaining the appropriate epigenetic pattern and transcription control at these promoters [37], [58]. Here, we explored the hypothesis that different latency types correspond to distinct chromatin conformations. Using 3C methodology, we found that different latency types form distinct chromatin-loops (Fig. 1 and 2). We identified two mutually exclusive loops that form between either OriP and Qp or OriP and Cp. These alternative loops correlate with promoter activity in their respective latency types. Genetic and siRNA depletion studies demonstrated that CTCF binding is essential for loop formation. We propose that chromosome conformation is an important epigenetic feature of latency type, and that CTCF plays a central role in generating EBV chromosome conformation (Fig. 9).

CTCF has been implicated in chromatin loop formation in multiple species and chromosome contexts [46]. CTCF binds upstream of Qp and Cp in both type I and type III latency, although with some reduced occupancy at Cp in type III latency [37]. Biochemical methods used in this study suggest that CTCF is intimately involved in loop formation between OriP and either Cp or Qp. Using the 3C-ChIP assay, we show that the CTCF at Cp is in close physical proximity to Qp in type I and to DS in type III (Fig. 4B). Similarly, we found that CTCF bound at Qp was in close proximity to DS in type I, but not in type III (Fig. 4C). We also observed that Qp contacts several other regions of the EBV genome, suggesting that EBV genome architecture may be more complex than a single loop between OriP and either Cp or Qp. It is also likely that CTCF links multiple sites in a hub to create more intricate chromatin structures, as has been shown in other model systems [46], [48]. The use of the 3C-ChIP assay may help to elucidate some of these more complex interactions within the EBV chromosome.

Genetic methods were also used to address the role of CTCF in EBV chromatin conformation (Figs. 5–7). EBV bacmids carrying a mutation in the CTCF binding sites at either Qp or Cp revealed a strong correlation between loop formation and promoter activation. Previously, we showed that the ΔCTCF-Qp bacmids have a decrease in Qp utilization and a corresponding increase in EBNA2 gene expression [37]. Here, using 3C methods, we found that ΔCTCF-Qp bacmids fail to form a loop between OriP and Qp, while Wt bacmids form these loops (Fig. 5C and 6A). Moreover, ΔCTCF-Qp bacmids formed an alternative loop between OriP and Cp, suggesting that the failure to form a type I conformation (Qp-OriP) resulted in a type III conformation (Cp-CTCF) (Fig. 5D). The biological implications of this finding are not immediately clear, but suggest that the natural evolution of EBV towards a type I latency requires the ability to form a loop between OriP and Qp, which is mediated by CTCF binding sites. Interestingly, CTCF binding at Cp was also essential for Qp-OriP loop formation, since CTCF-ΔCp bacmids failed to form the OriP-Qp loop, as well as the OriP-Cp loop (Fig. 5D and 6B). This suggests that CTCF (Cp)-CTCF (Qp) interactions may be important for long-distance loop formation between OriP and Qp (Fig. 9). Consistent with this model, siRNA depletion of CTCF led to a complete loss of all measurable loop formation at either Qp or Cp (Fig. 8), suggesting that CTCF is generally required for chromosome conformations measured at these loci.

Other factors, in addition to CTCF, must also contribute to loop formation and promoter selection. Components of OriP are known to contribute to viral chromosome conformation during latency. EBNA1, TRF2, and ORC bind to OriP in both type I and III latency, and have been implicated in chromosome structure [57], [60]. In experiments with extended cross-linking, we showed that EBNA1, TRF2, and ORC are in close proximity to the active promoter, namely Cp in type III and Qp in type I (Fig. 3). EBNA1 is known to form homotypic interactions through its linking regions, and is an essential transcription activator of Cp during B-cell immortalization [28], [61]. Although not directly tested in this study, it seems likely that EBNA1 will be required for loop formation between OriP and Qp. In some 3C experiments, we observed more efficient linkage of Cp to the DS region relative to the FR region (Fig. 2B). This may be due to the additional components that bind to DS, like TRF2 and ORC, or it may be due to the proximity and orientation of DS to Cp. However, EBNA1 is known to promote looping between DS and FR in vitro [62], [63], suggesting that OriP elements are likely to interact with each other and function coordinately in vivo. In addition, the FR can function as an EBNA1-dependent transcriptional enhancer [28], [29], [30], [64], [65]. Since our 3C data can not readily dissect the relative contributions of FR and DS to loop formation, we suggest that OriP functions as a single structural element that forms stable chromatin loops with either Cp in type III or Qp in type I latency.

Chromatin loop structure is likely to be an important epigenetic regulatory component of EBV latency gene expression. How these different loops and conformations are selected remains an important unanswered question. We have shown the CTCF is one essential component required for the establishment and maintenance of DNA loops that form between the OriP enhancer and the promoters at Cp and Qp. EBNA1, and components of OriP, must also be important for these interactions. It is also likely that other epigenetic modifications, including histone tail modifications and DNA methylation, contribute to loop formation, chromatin conformation, and promoter selection. DNA methylation of Cp is known to play a central role in the transcriptional silencing of Cp in type I latency [21], [34], [36], [58], [66], [67]. Furthermore, DNA methylation is known to inhibit CTCF binding at some sites, and the Cp CTCF binding site may be sensitive to changes in DNA methylation. In addition to changes in CTCF DNA binding, variation in CTCF protein levels may also contribute to promoter selection and loop formation [54]. CTCF is also known to be subject to several post-translational modifications, which may also contribute to selective loop formation [68], [69], [70]. Although the precise molecular mechanism of loop formation and promoter selection is not yet elucidated, our findings provide strong evidence that EBV chromatin conformation is an important structural component of latency type determination and, more generally, the establishment of stable epigenetic fates.

## Methods

### Cells

293 cells were cultured in Dulbecco's modified Eagle's medium (DMEM) supplemented with 10% fetal bovine serum and antibiotic in a 5% CO_2_ incubator at 37°C. EBV positive Mutu I and Mutu-LCL were cultured in suspension in RPMI 1640 medium supplemented with 10% fetal bovine serum and antibiotics in a 5% CO_2_ incubator at 37°C. Mutu-LCL were established by primary infection of peripheral blood mononuclear cells (PBMC) with EBV virions generated from stimulated Mutu I cells. 293-EBV Wt, ΔCTCF-Qp, and ΔCTCF-Cp have been previously described [37], [54] and were cultured as adherent cells in DMEM supplemented with 10% fetal bovine serum and 100 µg/liter hygromycin B in 5% CO_2_ incubator at 37°C. 293-EBV Wt carry a bacmid containing the EBV genome and express a green fluorescent protein (GFP) and hygromycin B drug resistance gene. 293-EBV ΔCTCF-Qp cells carry a bacmid containing the EBV genome mutated at the CTCF binding site at 50082 and express a green fluorescent protein (GFP) and hygromycin B drug resistance gene [37]. 293-EBV ΔCTCF-Cp cells carry a bacmid containing the EBV genome deleted at the CTCF binding site at 10393–10590 and express a green fluorescent protein (GFP) and hygromycin B drug resistance gene [54].

### Chromosome Conformation Capture (3C) assay

3C assay followed the protocol from Hagège et al. [71], with minor modifications. EBV-positive cells were filtered through a 70 µm to obtain a single cell preparation. 1×10^7^ cells were then fixed in 1% formaldehyde for 30 min at room temperature. 30 min cross-linking was found to be optimal since 10 min cross-linking resulted in few detectable 3C products (data not shown). The reaction was quenched with 0.125 M glycine and cells were collected by centrifugation at 230 g at 4°C. The pellet was resuspended in 0.5 ml cold lysis buffer (10 mM Tris-HCl, pH 7.5; 10 mM NaCl; 5 mM MgCl_2_; 0.1 mM EGTA) with freshly added 1× complete protease inhibitors (Roche) and was lysed on ice for 10 min. The nuclei were collected by centrifugation at 500 g for 10 min at 4°C and were resuspended in 0.5 ml of 1.4× Mse I buffer (New England Biolabs), including 0.3% SDS and were incubated for 1 h at 37°C while shaking at 1200 rpm. 2% (final concentration) Triton X-100 was added to the nuclei and then the samples were incubated for 1 hr at 37°C while shaking. 500 U Mse I (50,000 U/ml New England Biolabs) was added to the nuclei and the samples were incubated at 37°C over night while shaking. 10 µl of the samples were collected before and after the Mse I reaction to evaluate digestion efficiency. The reaction was stopped by addition of 1.6% SDS (final concentration) and incubation at 65°C for 30 minutes while shaking at 1200 rpm. The sample was then diluted 10 fold with 1.3× ligation Buffer (Roche) with added 1% Triton X-100 and was incubated for 1 h at 37°C while shaking at 900 rpm. 100 U T4 DNA ligase (5 U/µl Roche) were added to the sample and the reaction was carried at 16°C for 4 hrs followed by 45 min at room temperature. 300 µg of Proteinase K were added to the sample and the reaction was carried at 65°C overnight. RNA was removed by adding 300 µg of RNAse and incubating the sample for 1 h at 37°C. DNA was purified by twice phenol-chloroform extraction and ethanol precipitation. Purified DNA was then analyzed by conventional or quantitative PCR. As control for ligation products EBV Wt bacmid was digested with 10 U of Mse I overnight and then incubated with 10 U T4 DNA-ligase at 16°C overnight. The DNA was extracted by phenol-chloroform and precipitated with ethanol. Purified DNA was then analyzed by conventional or quantitative PCR. For Real time PCR, the ΔCt method was applied for analyzing data, using EBV-bacmid Ct values as control. Ct values were normalized for each primer pair by setting the Ct value of 100 ng of EBV bacmid control random ligation matrix DNA at a value of 1. Primer sequences for conventional PCR are listed in Table S1. Primer sequences for quantitative PCR are listed in Table S2.

### Chromatin Immunoprecipitation (ChIP) assay

ChIP assay followed the protocol provided by Upstate Biotechnology, Inc., with minor modifications as previously described [37]. Additional modifications are as follows. Cells were fixed in 1% formaldehyde for 30 minutes. DNAs were sonicated to between 200- and 350-bp DNA fragments on a Diagenode Bioruptor according to the manufacturer's protocol, and real-time PCR was performed with SYBER green probe in an ABI Prism 7000 using 1/100 to 1/2,500 of the ChIP DNA according to the manufacturer's specified parameters. Chromatin was immunoprecipitated using polyclonal antibodies for CTCF (Millipore), ORC2 (Pharmigen), EBNA1 (which was raised against full-length EBNA1), and TRF2 ( which was raised against full-length TRF2). The primers for amplification were as follows: DS (atgtaaataaaaccgtgacagctcat and ttacccaacgggaagcatatg), and OriLyt (gcccgttgggtttcattaag and ccaaatctcgcggacctcta) and Qp (ggctcacgaagcgagac and acaggacctgcgttatagcc) and Cp (gccgtggaaaaaaatttatgg and cgccaacaaggttcaattttct).

### Chromosome Conformation Capture Chromatin Immunoprecipitation (3C-ChIP) assay

EBV positive cells were fixed in 1% formaldehyde for 30 minutes at room temperature. The reaction was quenched by adding 0.125 M glycine (final concentration) and the cells were collected by centrifugation at 400 g for 5 min, washed twice with PBS and then 1×10^7^ were resuspended in 1 ml cold lysis buffer (1% SDS; 10 mM EDTA; 50 mM Tris-HCl, pH 8) with 1× complete protease inhibitors (Roche) and lysed on ice for 10 min. DNA was sonicated to between 200- and 350-bp DNA fragments on a Diagenode Bioruptor according to manufacturer's protocol. 100 µl sonicated cell lysate were then diluted 10 fold with immunoprecipitation (IP) buffer (0.01% SDS; 1.1% Triton X-100; 1.2 mM EDTA; 16.7 mM Tris-HCl, pH 8; 167 mM NaCl) and then pre-cleared by adding 50 µl 50% pre-blocked Protein A containing 25 µg of the heat-denaturated ssDNA and the samples were incubated for 30 min at 4°C with gentle rotation. The mixture was then centrifuged for 5 min at 750 g at 4°C and then the supernatant was carefully transferred into a new tube. Chromatin was precipitated by incubating the samples with 2.5 µg of antibody for CTCF (Millipore) or normal rabbit IgG (Santa Cruz) as negative control overnight at 4°C with rotation. Immune complexes were collected by incubation with 50 µl 50% slurry protein A sepharose beads (GE Healthcare) for 3 hrs at 4°C with rotation. Beads were collected by centrifugation at 800 g for 2 min and the washed once with 1 ml Low Salt buffer (0.1%SDS; 1% Triton X-100; 2 mM EDTA; 20 mM Tris-HCl, pH 8; 150 mM NaCl), then once with 1 ml High Salt buffer (0.1%SDS; 1% Triton X-100; 2 mM EDTA; 20 mM Tris-HCl, pH 8; 500 mM NaCl), then once with 1 ml LiCl buffer (250 mM LiCl; 1% NP-40; 1% Na-deoxycolate; 1 mM EDTA; 10 mM Tris-HCl, pH 8.0). Proteinase inhibitors cocktail (Sigma) was added to all the washing buffers before use. Before to eluting the immune complexes from beads we repaired the DNA ends generated by sonication in order to be ligated by T4 DNA ligation. The protein A beads were collected after the last wash by centrifugation at 800 g for 5 min and then resuspended into 100 µl kinase buffer [50 mM Tris-HCl, pH 7.5; 10 mM MgCl_2_; 1 mM DTT; 1 mM ATP; 35 µM dNTP; 6 U Klenow enzyme (Roche); 5 U T4 polynucleotide Kinase (Roche); 5 U T4 DNA polymerase (Roche)] and incubate for 90 minutes at 37°C with while shaking. The beads were centrifuged at 800 g for 3 minutes and then resuspended in 1 ml of 1× Ligation buffer (Roche) and 50 U T4 DNA ligase were added. The samples were then incubated at 16°C while shaking overnight. The beads were collected by centrifugation, washed twice with 1 ml TE and then resuspended in 150 µl Elution Buffer (1%SDS/TE) and incubated for 30 minutes at 65°C while shaking. The beads were centrifuged at 800 g for 5 minutes and the supernatant was carefully transferred into a new tube and incubated at 65°C overnight. Samples were then incubated with 45 µg Proteinase K for 3 hours at 50°C. After twice phenol/chloroform extraction and ethanol precipitation, purified DNA was amplified by nested PCR. For the first-round amplification we used High fidelity PlatinumTaq DNA Polymerase (Invitrogen) according to the manufacturer. The PCR conditions were the following: 94°C for 2 min followed by 35 cycles of 94°C for 30 s, 55°C for 30 s and 68°C for 1 min. For the second-round PCR 100 ng of first-round PCR DNA was used as template for Taq DNA polymerase (Roche). PCR conditions were the following: 94°C for 2 min followed by 30 cycles of 94°C for 30 s, 55°C for 30 s and 72°C for 1 min. Nested inverse primer sequences for both first and second round PCR are listed in Table S3. DNA generated by nested PCR was then analyzed by quantitative EBV-genome wide 384-well array.

### RNA extraction and RT-PCR

RNA was extracted from 5×10^6^ cells using the Qiagen RNA Extraction Kit according to the manufacturer's protocol (Qiagen). After extraction, the RNA was incubated with 2 U DNAse I at 37°C for 30 minutes, followed by the inactivation of the enzyme at 65°C for 10 minutes. The RNA was quantified and 2 µg of RNA was reverse transcribed using Super Script II Reverse Transcriptase from Invitrogen. 50 ng of cDNA was then analyzed by real time PCR, using the ΔCt method, using GFP gene as the internal control. Primer sequences are listed in Table S4.

### siRNA mediated knockout of CTCF

293 cells containing EBV bacmid, at the density of 2×10^6^ cells in 10 cm plate, were transfected with 50 pmol of short interfering RNA (siRNA) targeting CTCF (siD-020165-19) or non-targeting siRNA (si D-001810-01) (Dharmacon) as control, using DharmaFECT transfection reagent, according to manufacture's protocol. After 3 days post transfection cells were treated for 3C analysis as described above. Knockout efficiency was evaluated by western blot analysis.

## Supporting Information

Figure S1 **Primers validation for Real time PCR analysis of 3Cassay.** Primers used for Real time analysis of 3C assay were validated by agarose gel electrophoresis and ethidium bromide stain. EBV DNA bacmid digested with MseI and ligated were amplified used conventional PCR. PCR amplification products were visualized on 3% Nusieve agarose gel.
(TIF)

Figure S2 **Dissociation curve analysis for Qp anchor primer sets.** Dissociation curve analysis for Qp primer sets using EBV DNA bacmid digested with MseI and ligated was performed after a completed Real time PCR to exclude non specific products and primer dimers. Graph displays a plot of the first derivative of the rate of change in fluorescence as a function of temperature.
(TIF)

Figure S3 **Dissociation curve analysis for Cp anchor primer sets.** Dissociation curve analysis for Cp primer sets using EBV DNA bacmid digested with MseI and ligated was performed after a completed Real time PCR to exclude non specific products and primer dimers. Graph displays a plot of the first derivative of the rate of change in fluorescence as a function of temperature.
(TIF)

Figure S4 **Dissociation curve analysis for Control anchor primer sets.** Dissociation curve analysis for Control primer sets using EBV DNA bacmid digested with MseI and ligated was performed after a completed Real time PCR to exclude non specific products and primer dimers. Graph displays a plot of the first derivative of the rate of change in fluorescence as a function of temperature.
(TIF)

Figure S5 **Control for 3C-ChIP assay.** Samples were treated identically to 3C-ChIP assays shown in Fig. 4 with the exception of formaldehyde cross-linking which was excluded from these control reactions.
(TIF)

Figure S6 **Gene expression profile of 293-EBV wt and ΔCTCF bacmids.** Analysis of mRNA expression for EBNA1 and EBNA2 gene and promoter utilization by RT-qPCR. Data are normalized to GFP RNA and expressed as 2^−ΔCt^. Each bar represents the mean ± SE of three independent experiments.
(TIF)

Figure S7 **Control for 3C assays with bacmids.** Samples from Fig. 5 were assayed with anchor primers in the control region at 35401.
(TIF)

Figure S8 **Control for 3C assays with siRNA transfected bacmids.** Samples from Fig. 8 were assayed with anchor primers in the control region at 35401 by conventional PCR (A) and quantitative PCR (B).
(TIF)

Table S1 Primers sequence for conventional PCR analysis of 3C products.
(DOC)

Table S2 Primers sequence for real time PCR analysis of 3C products.
(DOC)

Table S3 Primers sequence for nested inverse PCR analysis of 3C-ChIP products.
(DOC)

Table S4 Primer list for RT-PCR.
(DOC)
