# Supplementary figures and images for: EBV Latency Types Adopt Alternative Chromatin Conformations
Source: PLoS Pathog. 2011 Jul 28;7(7):e1002180. doi: 10.1371/journal.ppat.1002180 (PMC3145795; doi:10.1371/journal.ppat.1002180)

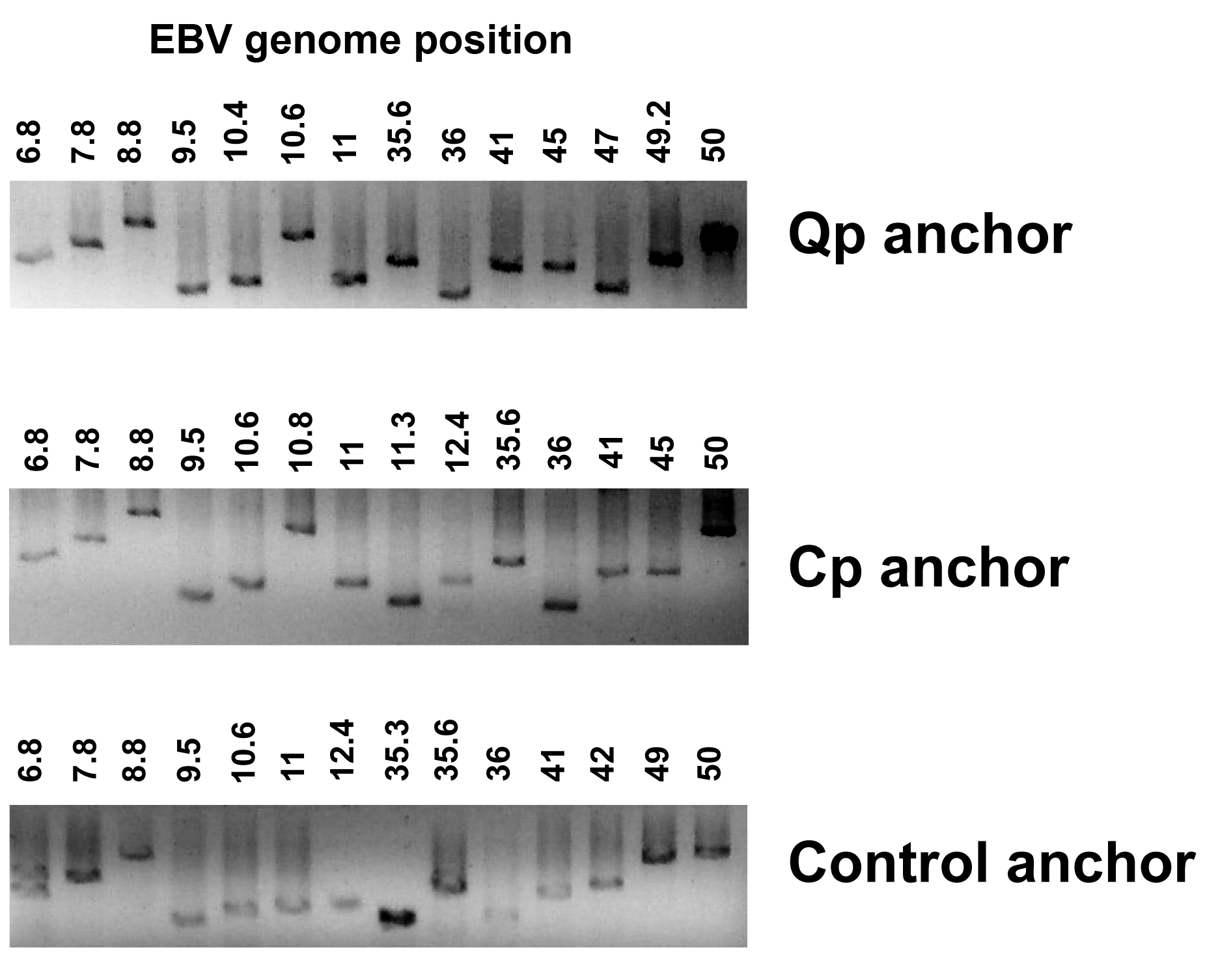

Supplement: Figure S1 — Primers validation for Real time PCR analysis of 3Cassay. Primers used for Real time analysis of 3C assay were validated by agarose gel electrophoresis and ethidium bromide stain. EBV DNA bacmid digested with MseI and ligated were amplified used conventional PCR. PCR amplification products were visualized on 3% Nusieve agarose gel. (TIF) [file ppat.1002180.s001.tif]

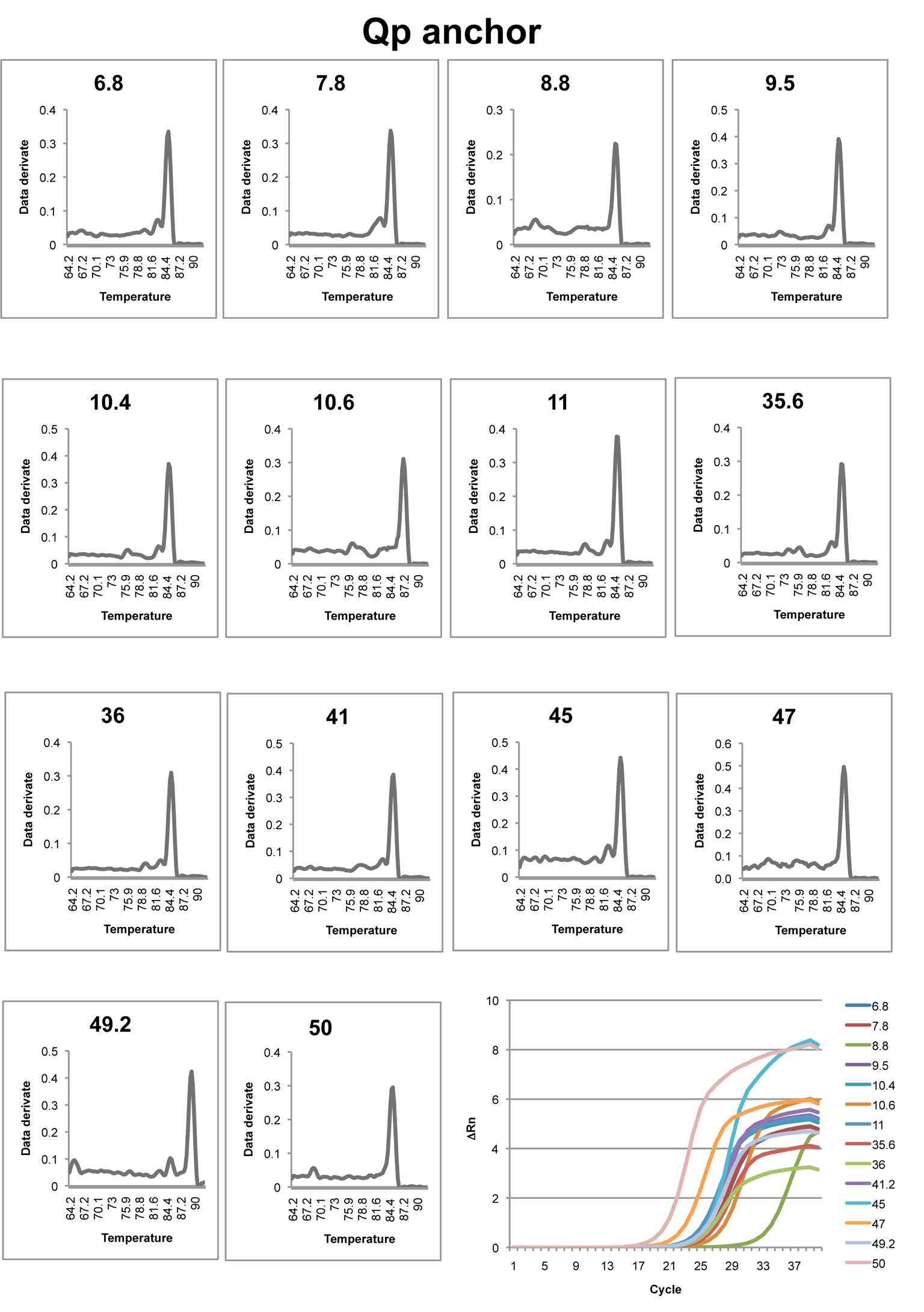

Supplement: Figure S2 — Dissociation curve analysis for Qp anchor primer sets. Dissociation curve analysis for Qp primer sets using EBV DNA bacmid digested with MseI and ligated was performed after a completed Real time PCR to exclude non specific products and primer dimers. Graph displays a plot of the first derivative of the rate of change in fluorescence as a function of temperature. (TIF) [file ppat.1002180.s002.tif]

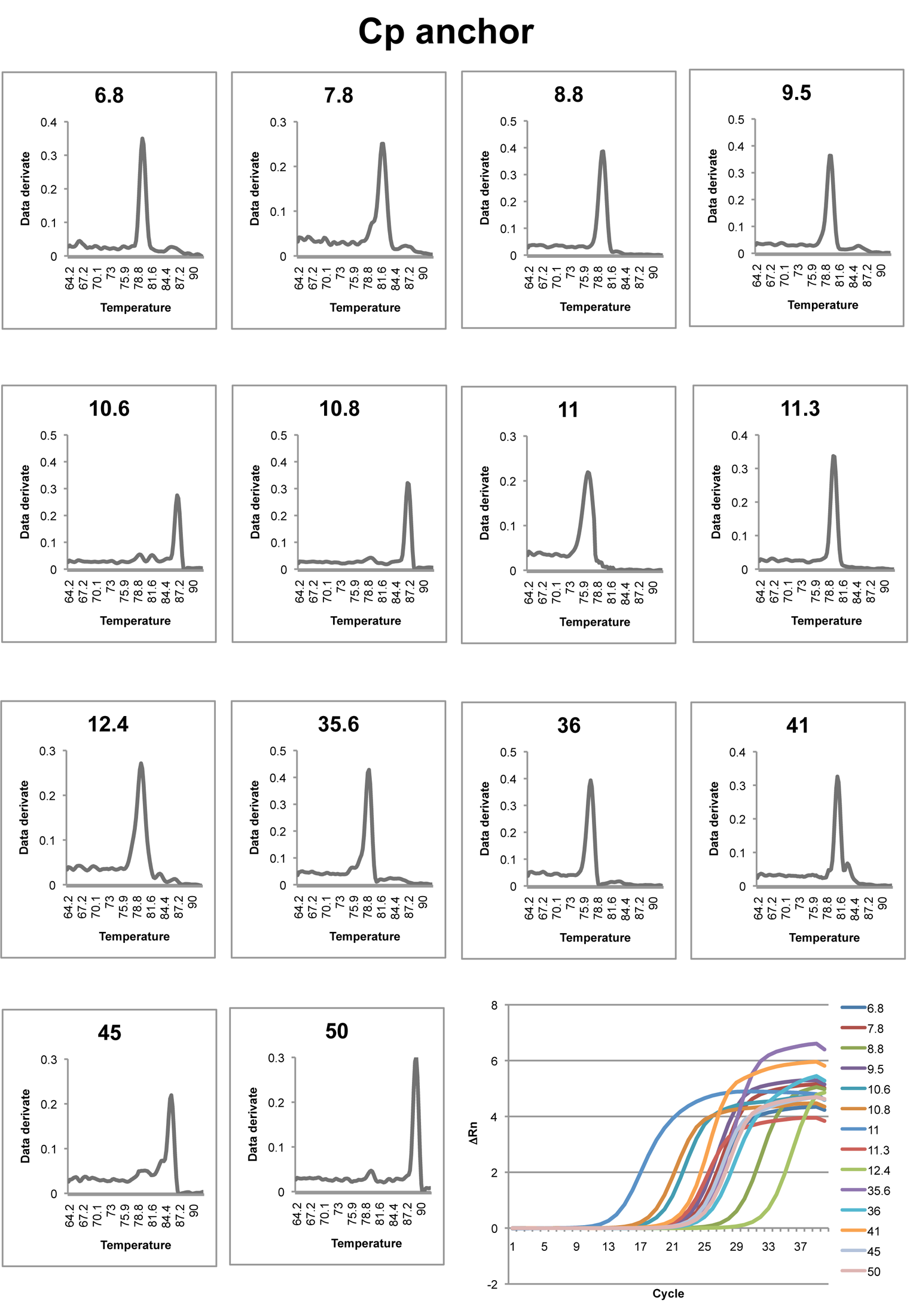

Supplement: Figure S3 — Dissociation curve analysis for Cp anchor primer sets. Dissociation curve analysis for Cp primer sets using EBV DNA bacmid digested with MseI and ligated was performed after a completed Real time PCR to exclude non specific products and primer dimers. Graph displays a plot of the first derivative of the rate of change in fluorescence as a function of temperature. (TIF) [file ppat.1002180.s003.tif]

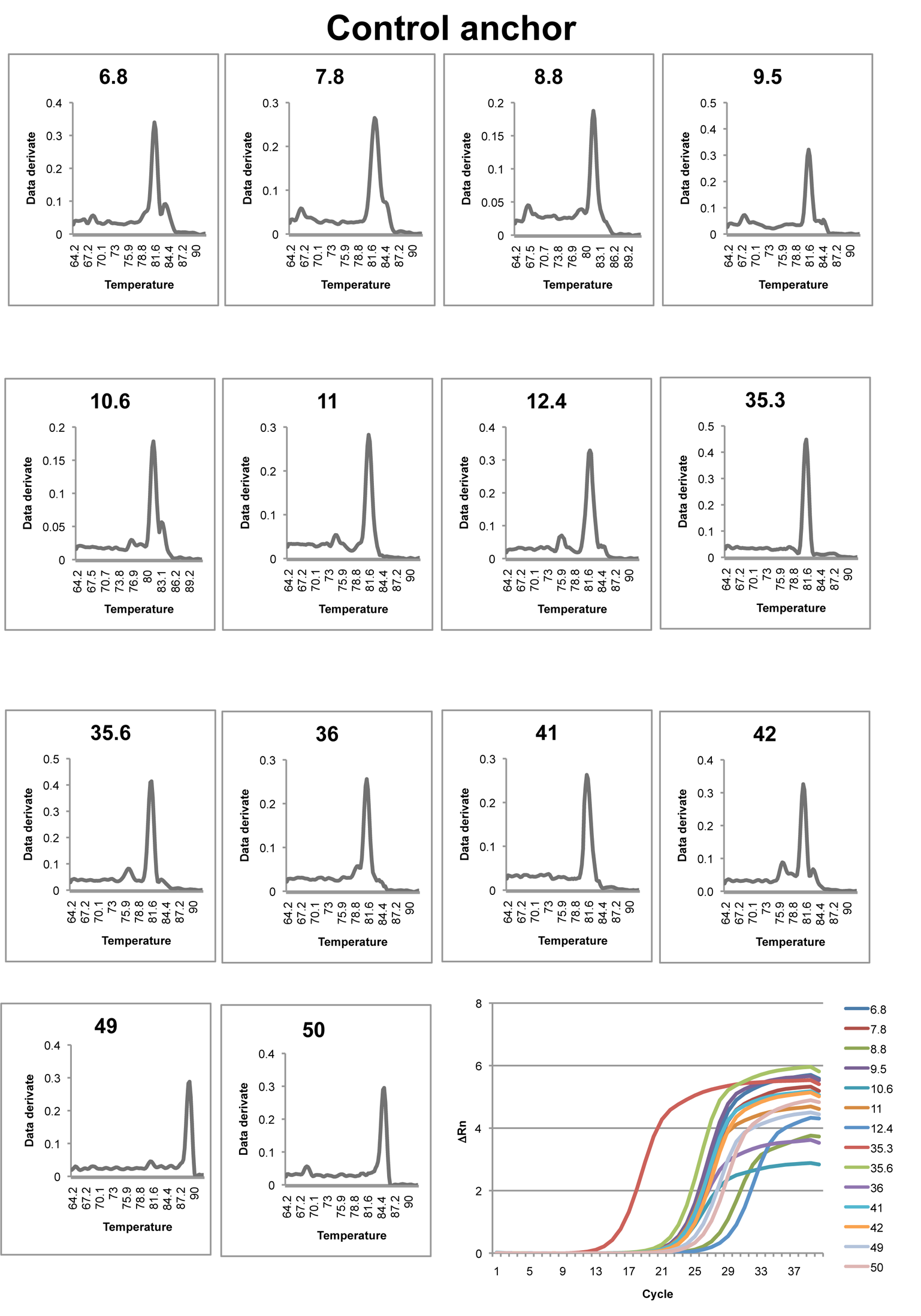

Supplement: Figure S4 — Dissociation curve analysis for Control anchor primer sets. Dissociation curve analysis for Control primer sets using EBV DNA bacmid digested with MseI and ligated was performed after a completed Real time PCR to exclude non specific products and primer dimers. Graph displays a plot of the first derivative of the rate of change in fluorescence as a function of temperature. (TIF) [file ppat.1002180.s004.tif]

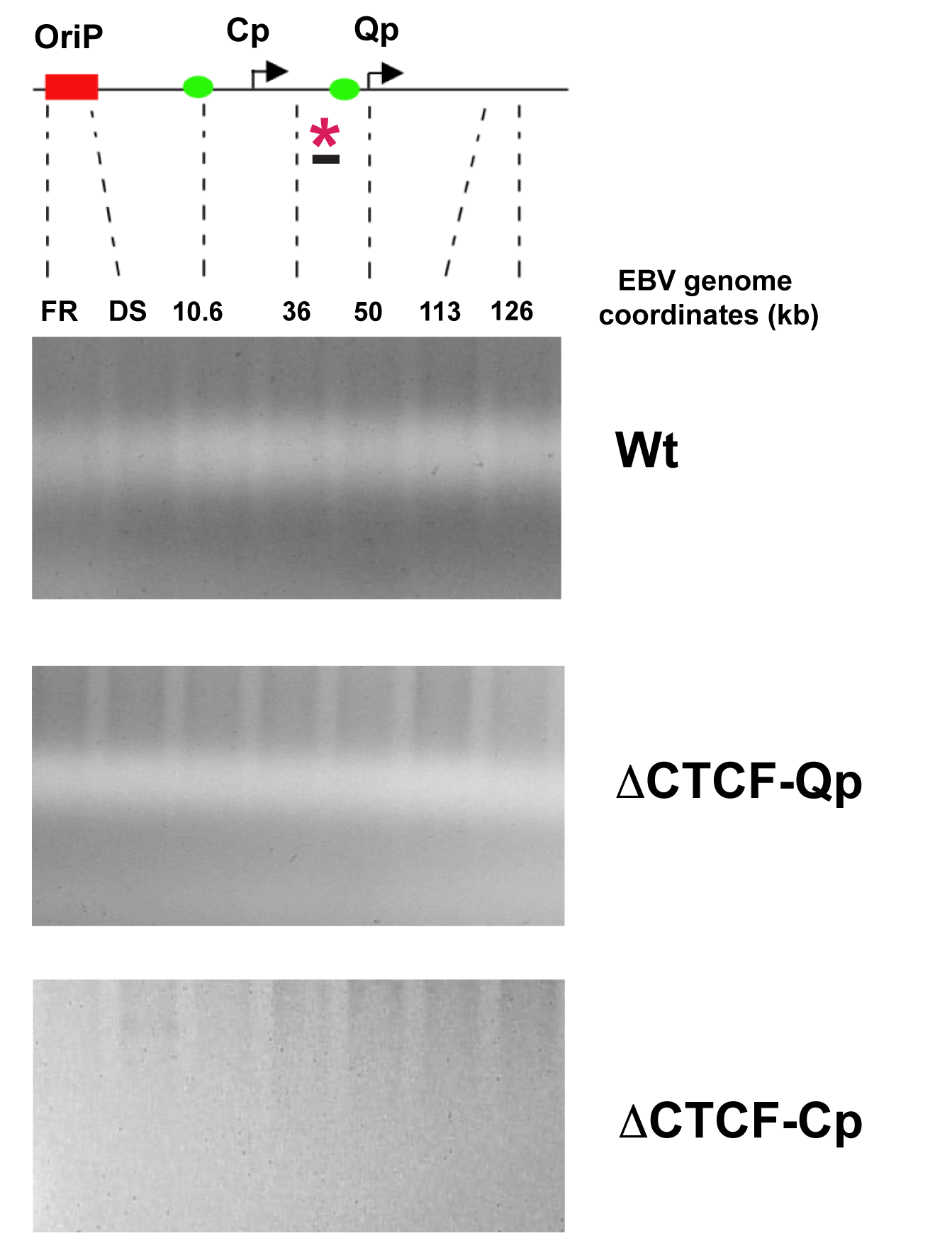

Supplement: Figure S5 — Control for 3C-ChIP assay. Samples were treated identically to 3C-ChIP assays shown in Fig. 4 with the exception of formaldehyde cross-linking which was excluded from these control reactions. (TIF) [file ppat.1002180.s005.tif]

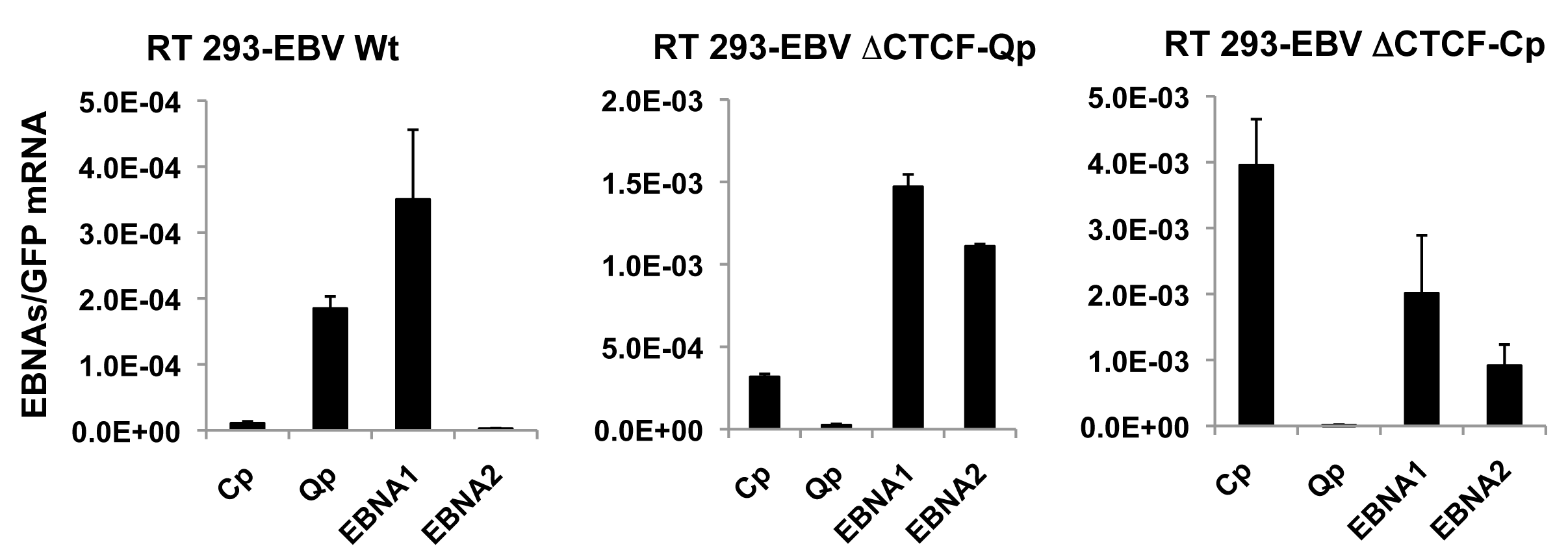

Supplement: Figure S6 — Gene expression profile of 293-EBV wt and ΔCTCF bacmids. Analysis of mRNA expression for EBNA1 and EBNA2 gene and promoter utilization by RT-qPCR. Data are normalized to GFP RNA and expressed as 2−ΔCt. Each bar represents the mean ± SE of three independent experiments. (TIF) [file ppat.1002180.s006.tif]

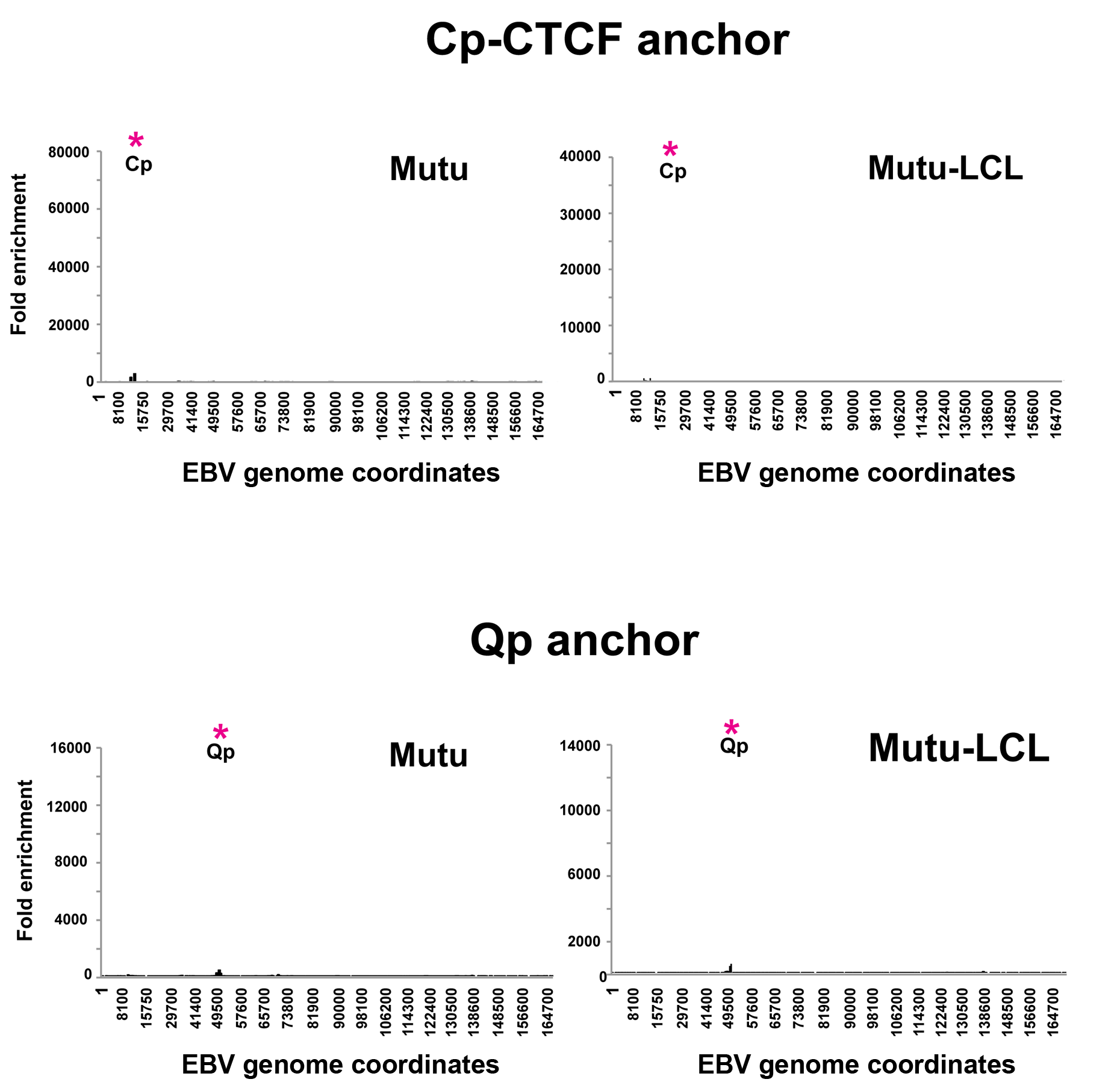

Supplement: Figure S7 — Control for 3C assays with bacmids. Samples from Fig. 5 were assayed with anchor primers in the control region at 35401. (TIF) [file ppat.1002180.s007.tif]

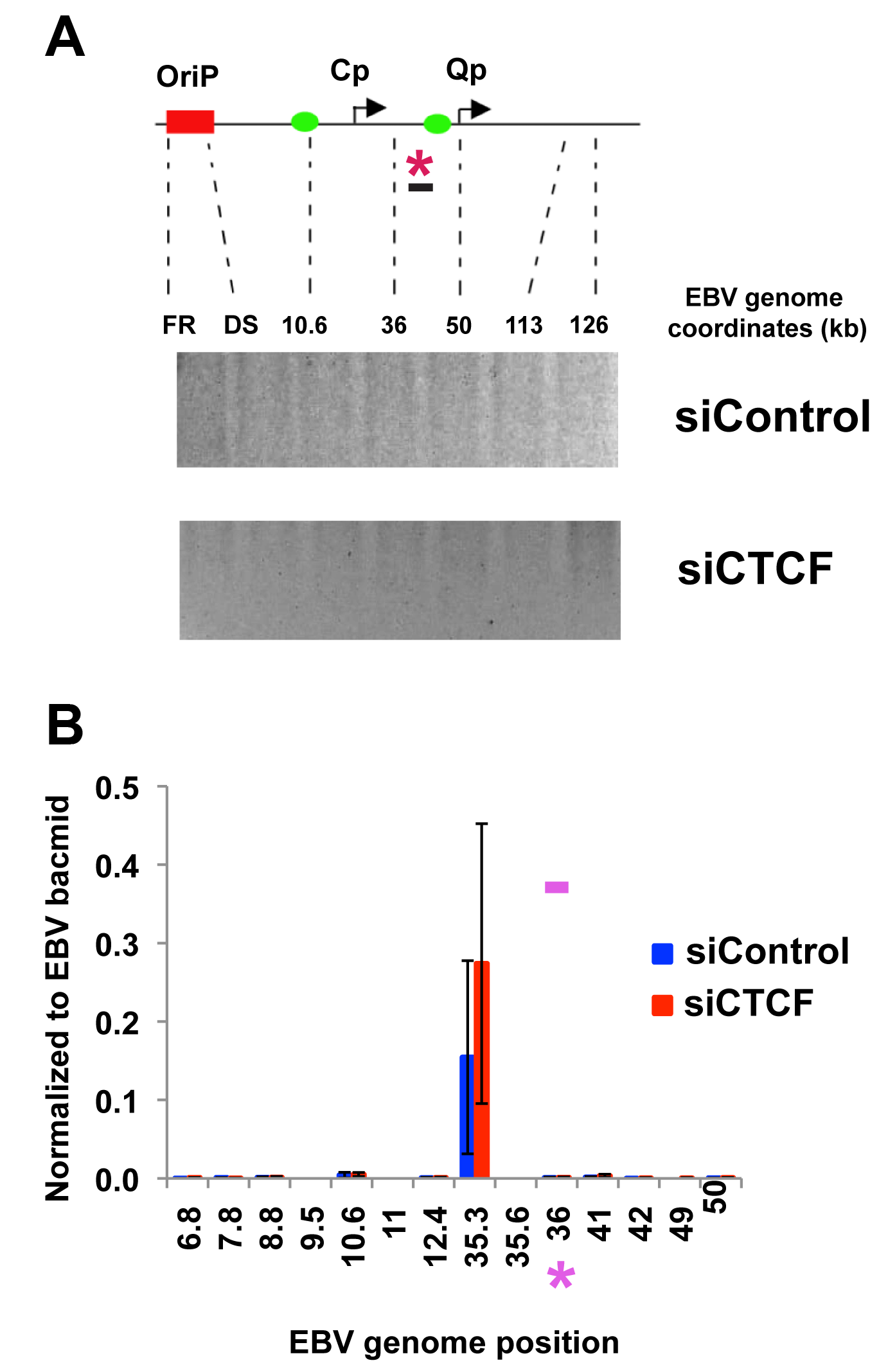

Supplement: Figure S8 — Control for 3C assays with siRNA transfected bacmids. Samples from Fig. 8 were assayed with anchor primers in the control region at 35401 by conventional PCR (A) and quantitative PCR (B). (TIF) [file ppat.1002180.s008.tif]
