# Supplementary material for: EBV Latency Types Adopt Alternative Chromatin Conformations
Source: PLoS Pathog. 2011 Jul 28;7(7):e1002180. doi: 10.1371/journal.ppat.1002180 (PMC3145795; doi:10.1371/journal.ppat.1002180)
Supplement: Table S1 — Primers sequence for conventional PCR analysis of 3C products. (DOC) [file ppat.1002180.s009.doc]

**Table 1.** Primers sequence for conventional PCR analysis of 3C product

| **Region** | **Sequence** | **Note** |
| --- | --- | --- |
| FR | TGTGTGTAATTTGTCCTCC |  |
| DS | AAACCGTGACAGCTCATG |  |
| 10.6 | TGCGAACAATTATTAGTAGC |  |
| Cp | TGGCTATAATCCGTCGCTCCTC | Anchor primer |
| 11 | TGGCGGGAGAAGGAATAACG |  |
| 11.3 | AGACAAGGACACCGAAGACC |  |
| Control | CTGCACATCTATAAACAGGC | Anchor primer |
| 36 | GGCTTGTGTTAGTGCTATG |  |
| 50 | GCTCACGAAGCCAGACAGTAC |  |
| Qp | AGGTGCGCCTATCCCAGTAC | Anchor primer |
| 113 | GACTATCCAGGTCTTGCC |  |
| 126 | ACAAGTAGCGGATGATAA |  |
