# Supplementary material for: EBV Latency Types Adopt Alternative Chromatin Conformations
Source: PLoS Pathog. 2011 Jul 28;7(7):e1002180. doi: 10.1371/journal.ppat.1002180 (PMC3145795; doi:10.1371/journal.ppat.1002180)
Supplement: Table S2 — Primers sequence for real time PCR analysis of 3C products. (DOC) [file ppat.1002180.s010.doc]

**Table 2.** Primers sequence for real time PCR analysis of 3C product

| **Region** | **Sequence** |  |
| --- | --- | --- |
| 6.8 | GTGAGGACGGTGTCTGTGGTT |  |
| 7.8 | GGCCCGCCCACCTACTT |  |
| 8.8 | GCACAATGCCACCACTGAAC |  |
| 9.5 | CCCAAATGTTGAGGGACCTAAG |  |
| 10.4 | ACCCGACCCAGCCACTTAC |  |
| 10.6 | CACCTGCAGCCTACAAAAGTACA |  |
| 10.8 | TTAGAAACCCAAGCGCAGAAA |  |
| 11 | GCCCCGTGGGACCTTAGA |  |
| 11.3 | TCATCGCAGGGTTCTTACCAT |  |
| 12.4 | CCCAAACTGGGCTTCAGATG |  |
| 35.3 | CGCCCAAGCTGCTTTG |  |
| 35.6 | CCAGCGCCAATCTGTCTACATA |  |
| 36 | CCAGGTGGCAGCCTGTTTA |  |
| 41.2 | CCTGCCTCACCATGACACACT |  |
| 45 | TCTCGCAGAGTGGGCAGAT |  |
| 47 | TGGGTCTCTCAACGGATGTTG |  |
| 49.2 | AGTACGTGCAGAGGACTTTTGG |  |
| 50 | GCTCACGAAGCCAGACAGTAC |  |
| Cp | GCCCCGCCTGGTGTTATTA | Anchor |
| Qp | TCCCAGCTGCCCAAAATG | Anchor |
| Control | TTGGCGCTGGGTGGTT | Anchor |
