# Supplementary material for: EBV Latency Types Adopt Alternative Chromatin Conformations
Source: PLoS Pathog. 2011 Jul 28;7(7):e1002180. doi: 10.1371/journal.ppat.1002180 (PMC3145795; doi:10.1371/journal.ppat.1002180)
Supplement: Table S3 — Primers sequence for nested inverse PCR analysis of 3C-ChIP products. (DOC) [file ppat.1002180.s011.doc]

**Table 3.** Primers sequence for nested PCR analysis of 3CIP products

| **Name** | **Sequence** | **Region** |
| --- | --- | --- |
| Qp 1 st round Fw | AAGTGCTTGAAAAGGCGCGGG | 50126-50146 |
| Qp 1 st round Rev | TCTGGCCTCTGTCCGCAAAG | 49082-49063 |
| Qp 2 nd round Fw | GCGGGTAATACATGCTATC | 50167-50189 |
| Qp 2 nd round Rev | AAGTTAAATACAGGAGCTGC | 49065-49046 |
| Cp 1 st round Fw | CCTTGCGAACAATTATTAGTAG | 10738-10759 |
| Cp 1 st round Rev | AGTGGCTGGGTCGGGTAATC | 10404-10385 |
| Cp 2 nd round Fw | AAGCGCAGAAATTAGTTG | 10862-10879 |
| Cp 2 nd round Rev | GGAGAACACAGGAAGGTAC | 10327-10309 |
