# Supplementary material for: EBV Latency Types Adopt Alternative Chromatin Conformations
Source: PLoS Pathog. 2011 Jul 28;7(7):e1002180. doi: 10.1371/journal.ppat.1002180 (PMC3145795; doi:10.1371/journal.ppat.1002180)
Supplement: Table S4 — Primer list for RT-PCR. (DOC) [file ppat.1002180.s012.doc]

**Table 4**. Primer list for RT-PCR

| **Name** | **Sequnece** |
| --- | --- |
| Cp transcrip fw | TGCCTGAACCTGTGGTTGG |
| Qp transcript fw | GTGCGCTACCGGATGGC |
| Cp/Qp transcript rev | CATGATTCACACTTAAAGGAGACGG |
| EBNA1 fw | GGTCGTGGACGTGGAGAAAA |
| EBNA1 rev | GGTGGAGACCCGGATGATG |
| EBNA2 fw | GCTTAGCCAGTAACCCAGCACT |
| EBNA2 rev | TGCTTAGAAGGTTGTTGGCATG |
| GFP fw | AGCAAAGACCCCAACGAGAA |
| GFP rev | GGCGGCGGTCACGAA |
